# Interaction Between Zn Deficiency, Toxicity and Turnip Yellow Mosaic Virus Infection in *Noccaea ochroleucum*

**DOI:** 10.3389/fpls.2020.00739

**Published:** 2020-06-05

**Authors:** Filis Morina, Archana Mishra, Ana Mijovilovich, Šárka Matoušková, Dennis Brückner, Josef Špak, Hendrik Küpper

**Affiliations:** ^1^Department of Plant Biophysics and Biochemistry, Biology Centre, Institute of Plant Molecular Biology, Czech Academy of Sciences, České Budějovice, Czechia; ^2^Department of Geological Processes, Czech Academy of Sciences, Institute of Geology, Rozvojová, Czechia; ^3^Deutsches Elektronen-Synchrotron DESY, Hamburg, Germany; ^4^Department of Physics, University of Hamburg, Hamburg, Germany; ^5^Faculty of Chemistry and Biochemistry, Ruhr-University Bochum, Bochum, Germany; ^6^Department of Plant Virology, Biology Centre, Institute of Plant Molecular Biology, Czech Academy of Sciences, České Budějovice, Czechia; ^7^Department of Experimental Plant Biology, University of South Bohemia, České Budějovice, Czechia

**Keywords:** cadmium, chlorophyll fluorescence kinetics, metal transporters, non-hyperaccumulator, plant immunity, TYMV infection, zinc, micro X-ray fluorescence

## Abstract

Zinc is essential for the functioning of numerous proteins in plants. To investigate how Zn homeostasis interacts with virus infection, Zn-tolerant *Noccaea ochroleucum* plants exposed to deficient (Zn’0’), optimal (Zn10), and excess Zn (Zn100) concentrations, as well as Cd amendment, were infected with Turnip yellow mosaic virus (TYMV). Imaging analysis of fluorescence kinetics from the μs (OJIP) to the minutes (Kautsky effect, quenching analysis) time domain revealed strong patchiness of systemic virus-induced photosystem II (PSII) inhibition. That was more pronounced in Zn-deficient plants, while Zn excess acted synergistically with TYMV, in both cases resulting in reduced PSII reaction centers. Infected Cd-treated plants, already severely stressed, showed inhibited non-photochemical quenching and PSII activity. Quantitative *in situ* hybridization at the cellular level showed increased gene expression of *ZNT5* and downregulation of *HMA4* in infected Zn-deficient leaves. In Zn10 and Zn100 infected leaves, vacuolar sequestration of Zn increased by activation of *HMA3* (mesophyll) and *MTP1* (epidermis). This correlated with Zn accumulation in the mesophyll and formation of biomineralization dots in the cell wall (Zn100) visible by micro X-ray fluorescence tomography. The study reveals the importance of adequate Zn supply and distribution in the maintenance of photosynthesis under TYMV infection, achieved by tissue-targeted activation of metal transporter gene expression.

## Introduction

Zinc availability is among the most common nutrient factors that limit plant yield and food quality. The role of Zn as an essential nutrient in overall metabolism and throughout plant development has been well established. Besides being a cofactor of various enzymes, Zn is involved in transcriptional regulation, and it is essential for plant sensing and response to environmental stimuli (Krämer and Clemens, 2005; Broadley et al., 2007; Andresen et al., 2018).

However, when present in excess, Zn is detrimental for plants. Mechanisms for maintaining Zn homeostasis include efflux, sequestration and binding to ligands. Plant requirements for Zn are species-specific and range from low to exceptionally high as in the case of hyperaccumulators like *Noccaea* (formerly *Thlaspi) caerulescens* and *Arabidopsis halleri*. Both species are closely related to *A. thaliana*, which makes them attractive models to study mechanisms of Zn tolerance and accumulation, as well as its functions. The evolutionary advantage of metal hyperaccumulation was explained as a defense mechanism against pathogenic bacteria (Fones et al., 2010) and herbivores [reviewed by Boyd (2012), Leitenmaier and Küpper (2013), Stolpe et al. (2017)]. On the other hand, the joint effects hypothesis suggests synergistic effects of metal accumulation and organic defense against biotic stress, and a cross-talk between these pathways has been proposed (Harada et al., 2010; Kazemi-Dinan et al., 2015; Gallego et al., 2017). Nevertheless, if and how Zn content correlates with virus resistance has not been studied extensively, especially in non-hyperaccumulator plants. One of the rare studies, involving the Ni hyperaccumulator *Streptanthus polygaloides* and the non-hyperaccumulator *S. insignis*, showed higher susceptibility to Turnip mosaic virus at high Ni compared to low Ni in both species (Davis et al., 2001).

Here, we used the non-hyperaccumulator *Noccaea ochroleucum*, a species closely related to *N. caerulescens*, and Turnip yellow mosaic virus (TYMV) as a model of a common virus that replicates in association with the chloroplast outer membrane. The aim was to reveal the interaction between Zn accumulation and distribution with the virus infection. The Zn accumulation capacity of *Noccaea ochroleucum* (at that time still called *Thlaspi ochroleucum*) in comparison to *Noccaea (Thlaspi) caerulescens* has been described by Shen et al. (1997) and McGrath et al. (1997). The tolerance of both species to Cd and Zn has also been compared in our own earlier work (Küpper et al., 2007a). Although a non- hyperaccumulator, *N. ochroleucum* can still accumulate relatively high Zn contents in the shoots compared to crop plants or *Arabidopsis thaliana*; therefore it is a suitable species for investigating Zn distribution (Mijovilovich et al., 2019) and gene expression of Zn transporters on the cellular level.

TYMV is specific to the Brassicaceae family. It is easily transmitted by mechanical inoculation, and it can efficiently induce systemic infection. Another advantage of using TYMV as a model for plant-virus interactions is that it is not lethal for plants, which enables studying simultaneously plant acclimation to nutrient and virus stress in long term experiments. Seed transmission of TYMV has been described for the first time in oilseed and turnip by Špak et al. (1993). TYMV is inducing a stable systemic infection in *Brassica rapa*, subsp. *pekinensis* reducing the amount of pigments in the plant by inhibition of their synthesis with a moderate impact on growth (Crosbie and Matthews, 1974). Symptoms of TYMV infection are chlorotic local lesions and systemic yellow mosaic patches. TYMV replication is facilitated by numerous vesicular invaginations of the outer chloroplastic membrane (Prod’homme et al., 2001). Infection results in formation of clusters made of swollen and vacuolized chloroplasts with increased number of plastoglobuli. Other infection symptoms are reduced photosynthesis, lipid peroxidation and ROS generation, along with transcriptomic, proteomic and metabolomic changes in infected chloroplasts (Bilgin et al., 2010; Li et al., 2016).

The hypothesis for the current study was that plant response to virus infection may be modulated by Zn tissue-distribution and by Zn availability for primary metabolic processes. The hypothesis is based on the essential role of Zn as a cofactor of enzymes, such as carbonic anhydrase involved in photosynthesis and superoxide dismutase (CuZnSOD) involved in ROS regulation, as well as the regulatory role of zinc finger proteins in numerous signaling processes under abiotic and biotic stress (Talke et al., 2006; Ciftci-Yilmaz and Mittler, 2008; Andresen et al., 2018; Noman et al., 2019). Thus, we aimed to investigate the targeted distribution of Zn as defense response to TYMV. Plants were grown under Zn deficient, optimal and excessive Zn conditions, as well as in the presence of Cd as a non-essential toxic metal, which shares the mechanisms of uptake and sequestration with Zn (Andresen and Küpper, 2013).

In this study, we used a recently introduced improved technique for accurate analysis of ultra-fast measurements of chlorophyll fluorescence kinetics, OJIP (Küpper et al., 2019). This was combined with analysis of subcellular metal distribution by micro X-ray fluorescence tomography on frozen-hydrated samples and with analysis of gene expression of metal transporters on the whole leaf level by real-time PCR as well as on the tissue level by quantitative *in situ* hybridization, QISH (Küpper et al., 2007b) with the aim to correlate TYMV effects on photosynthesis with Zn transport and distribution. For comparison, *HMA3*, *HMA4*, *MTP1*, and *ZNT5* transporters were chosen according to the previous reports on their role in the metal accumulation and sequestration in *N. caerulescens* [review by Küpper and Kochian (2010), Mishra et al. (2017), Andresen et al. (2018)]. *HMA3* and *HMA4* are closely related members of the P_1__*B*_-type ATPase family; *HMA3* is located in the tonoplast and actively sequesters Zn into the vacuole, while *HMA4* is responsible for xylem loading in the roots and stems, and efflux toward the apoplast in the shoots (Verret et al., 2004). *MTP1* is another vacuolar transporter increasing Zn concentration in the leaves and directing it to specific shoot tissues, e.g. vasculature and epidermal storage cells, while the plasma membrane transporter *ZNT5* has been involved in Zn accumulation in epidermal metal storage cells (Küpper and Kochian, 2010; Sinclair and Krämer, 2012).

## Materials and Methods

### Plant Cultivation and Experimental Set-Up

*Noccaea ochroleucum* (J. Presl and C. Presl) F.K. Mey. (formerly *Thlaspi ochroleucum* J. Presl and C. Presl) seeds were given to us in 1999 by colleagues who had characterized this species (McGrath et al., 1997; Shen et al., 1997). The seeds originated from a population on Thasos, Greece. Our colleagues characterized them in terms of metal uptake in comparison to *Noccaea caerulescens* (at that time still called *Thlaspi caerulescens*). We had earlier compared metal tolerance and long-term metal toxicity stress in these two species (Küpper et al., 2007a). This species is not self-compatible, and it was propagated by self-pollination. For stratification, the seeds were spread on moistened perlite: vermiculite (3:1) in glass dishes and incubated at 4°C in a refrigerator for 2 weeks, followed by germination at 20–25°C in a greenhouse. The 3 weeks old seedlings were transferred into a controlled environment chamber, where they were grown in 2.5 L plastic pots (four plants per pot) filled with nutrient solution containing either 0.01 (in the further text labeled as Zn’0’), 10, 100 μM Zn or 10 μM Zn+1 μM Cd in duplicates (Küpper et al., 2007a). The nutrient solution was continuously aerated using a lab-built system (Küpper et al., 2007a) and automatically renewed with a flow rate of 250 ml.d^–1^.plant^–1^ by a programmable peristaltic pump (Ismatec MCP process, Cole-Parmer GmbH, Wertheim, Germany). The growth chamber was maintained at 14 h day length and 22°C (day)/18°C (night) temperature. The photon flux density during the light period followed an approximately sinusoidal cycle with a maximum of around 150 μmol.m^–2^.s^–1^ and was supplied with full-spectrum discharge lamps. After 8 weeks, half of the plants were used for infection, and the other half was treated as non-infected controls. Three months after the start of infection, the leaves were used for *in vivo* measurements, and the fresh shoot biomass (leaf rosette) was measured. This period was chosen to ensure systemic virus infection of the plants and to determine long-term effects of virus exposure. Two independent experiments were performed.

### Virus Infection Procedure

The culture of TYMV (*Turnip yellow mosaic virus*) was maintained on Chinese cabbage (*Brassica rapa*, subspecies *pekinensis*) plants under conditions described above. The virus culture was renewed every 3 months to maintain the same age of the culture on its host. Virus inoculum was prepared by homogenizing a small amount (about 200 mg) of infected leaf tissue (Chinese cabbage fully developed leaves) in phosphate buffer (pH 7.4). Eight weeks after transfer to hydroponic solution and start of the metal treatment, a pair of healthy *N. ochroleucum* leaves was chosen for inoculation. Typically, the 2nd or 3rd pair of the youngest leaves was dusted with carborundum powder, and the virus homogenate was applied by gently stroking it onto the leaf surface with a sterile cotton bud. Control plants received the same treatment, but only with the phosphate buffer (pH 7.4).

### Fluorescence Kinetic Microscopy Measurements

Chlorophyll fluorescence kinetics was measured 3 months after the start of virus infection. Fully expanded leaves (one leaf per plant, four leaves per treatment in each experiment) were inserted into a measuring chamber, covered with a slightly moist cotton pad and fixed by a mesh, and aerated by water-saturated room air using a pump (Küpper et al., 2007a). Changes in biophysical parameters of the photosynthetic light reactions were determined using Fluorescence Kinetic Microscopy (FKM). At first, the fast rise from F_0_ to F_ m_ was measured as an OJIP transient by a newly developed system (Küpper et al., 2019). Then the slower kinetics (classical quenching analysis, Kautsky effect) was measured using the FKM built earlier (Küpper et al., 2007a). For the OJIP measurements, the irradiance of actinic light was 675 μmol.m^–2^.s^–1^. The length of each measuring light flash, and the synchronized frame of the camera was 20 μs. After measuring F_0_ as an average of five pictures, a 2100 ms period of actinic light was given to record the OJIP kinetics as described by Küpper et al. (2019).

FKM measurements of the Kautsky effect were done using the same instrument and protocol as described in detail by Küpper et al. (2007a) with modifications by Andresen et al. (2010). In summary, the 5 min dark-acclimated plants were treated with a supersaturating flash (590 μmol.m^–2^.s^–1^) for measurement of maximal dark-adapted fluorescence quantum yield (F_ m_), followed by 200 s dark phase, at the end of which the minimal dark-adapted fluorescence quantum yield (F_0_) was measured (0.5 μmol.m^–2^.s^–1^), then 200 s actinic light (45 μmol.m^–2^.s^–1^) phase was applied, and finally, 200 s relaxation was given in the dark. In the actinic light and relaxation phases, further supersaturating flashes were applied for measurements of PSII activity (Φ_ PSII_) and regulation of non-photochemical energy dissipation (NPQ).

For all the chlorophyll fluorescence measurements, the data are averages of two independent experiments. All microscopic measurements were done on the mesophyll away from the veins. Three mesophyll areas on each leaf were chosen for analysis.

The parameters chosen to describe the quantum yields and efficiencies/probabilities of trapped electrons to be transported through the photosystems [according to Stirbet and Govindjee (2011)], and parameters for the Kautsky effects are shown in Supplementary Table S1. For OJIP measurements, the checkpoints were chosen by time. All imaging fluorescence kinetic records were analyzed using the FluorCam 7 software (Photon Systems Instruments, Brno, Czech Republic) as described by Küpper et al. (2019).

### RNA Extraction and Quantitative Real-Time PCR Analysis

For all RNA isolations, TRIzol reagent (Ambion technologies, United States) was used according to manufacturer’s instructions. RNA quality was confirmed using a Nanodrop 100 spectrophotometer (Thermo Fisher Scientific, United States). RNA was purified from any remaining DNA with DNase I (RQ1 RNase-Free DNase, Promega, United States) according to the manufacturer’s instructions. After treatment with DNase I, cDNA was synthesized using the Maxima First Strand cDNA Synthesis Kit for RT-qPCR (Cat. No.: 1641; Biogen) according to the supplier’s instructions using 1 μg RNA per reaction. Since the genome of *N. ochroleucum* is unknown and the information on gene copy numbers is not available for this species, we used primer pairs for *N. caerulescens* (as a closely related species). Primers for *HMA3* and *HMA4* were previously designed (Mishra et al., 2017), while primers for *ZNT5* and *MTP1* genes were designed in the current study. Preliminary experiments showed that the primers designed for *N. caerulescens* were suitable for *N. ochroleucum*, because all these primers were designed to anneal to conserved regions of the genes investigated. For the same reason, PCR with these primers amplifies the total number of transcripts from all transcribed copies of these genes in the genome.

To verify that all plants were infected with the virus, RNA was extracted from the leaves of 2-months-old plants (one leaf per plant). The virus cDNA was amplified by PCR as described previously (Penazova et al., 2016) and visualized on the 1% agarose gel using GelRedAgarose LE (Biotium). Primer sequences are shown in Supplementary Table S2.

For measuring mRNA abundance of metal transporters, total RNA was extracted 3 months after the start of TYMV infection from fully developed leaves of *N. ochroleucum* (from two independent experiments, and 3–4 plants in each experiment). Real-time PCR was carried out with a Q-Tower 3 (Analytik Jena, Germany) in a total volume of 14 μl including 7.5 μl of MasterMix (Xceed SG qPCR 2x Mix Lo ROX), 1 μl of x10 diluted cDNA, 0.3 μM of reference genes (*GAPDH* and *18S*) and 0.6 μM of each gene-specific primer (Supplementary Table S2). The reference genes that were used for normalization, *GAPDH* and *18S* RNA are described in Mishra et al. (2017). The Ct variation of the samples for the reference genes was about 5% between non-infected and infected plants. Running qPCR conditions were as follows: initial treatment (3 min, 95°C), denaturation (10 s, 95°C), annealing (30 s, 56°C), extension (15 s, 60°C) for 40 cycles. The reaction was stopped by 30 s at 95°C. Product specificity was tested by melting curve analyses (65–95°C). QPCR efficiencies were checked in all cases, and they were between 90 and 115%. Data collection was performed with at least three different measurements per sample. Pfaffl (2001) method was used to calculate the expression rate and standard deviation (SD) between the target group and the control group.

### Quantitative mRNA *in situ* Hybridization

The analysis of gene expression of metal transporters in different tissues was done using a method for quantitative mRNA *in situ* hybridization (QISH) developed by Küpper et al. (2007b). Briefly, the young-mature leaves of *N. ochroleucum* plants from different treatments (3 months after start of infection) were cut into 2 × 3 mm pieces. The samples were fixed with paraformaldehyde and hybridized with fluorescently labeled probes: *HMA3*, *HMA4*, *MTP1*, and *ZNT5* for the genes of interest (GOI) and cytosolic *GAPDH* as a reference gene. The probes (fluorescently labeled DNA oligonucleotides) were designed to have identical melting temperatures and GC content and no problematic features like self-dimerization. The fluorescent label Cy5 was used for the GOI and Bodipy-TMR-X for the *GAPDH*, for the reasons described in detail by Küpper et al. (2007b). QISH image processing was done as in Küpper et al. (2007b), using ImageJ 1.8.0_112 software. The data from one experiment using fully developed leaves from three to four plants were analyzed. Confocal microscopy measurements were performed as described by Küpper et al. (2007b).

### Analysis of Zn Distribution in Leaves Using Micro X-Ray Fluorescence Tomography

Sample preparation, μXRF measurements and data analyses were done as described in detail by Mishra et al. (2016). Briefly, we used the μXRF beamline P06 (storage ring PETRA III, DESY, Hamburg, Germany) to analyze shock-frozen-hydrated samples mounted in polyimide capillaries of 1 mm diameter in a cryostream combined with the Maia detector (Boesenberg et al., 2016). Data reduction was performed with the GeoPIXE software, and tomographic reconstruction was done via the maximum-likelihood expectation-maximization (MLEM) algorithm with a customized script software, quantification and absorption correction were done with multielement standards that were prepared and recorded as tomograms like the samples. Multielement standards were prepared in 10% glycerol containing all elements of interest. We analyzed at least three replicates from two independent experiments of each of the following sample types: leaves from plants grown at replete Zn concentration (10 μM Zn^2+^), and sublethally toxic Zn concentration (100 μM Zn^2+^). For both metal treatments, TYMV virus-infected and non-infected leaves were analyzed.

### Element Analysis by ICP-MS

For ICP-MS analysis, leaves were frozen in liquid nitrogen and freeze-dried (one leaf per plant, four leaves per treatment in each experiment). About 50 mg of homogenized freeze-dried tissue was weighed in glass tubes. All glassware used had previously been acid-washed with 5% HNO_3_. A mixture of 85 ml/100 ml 70% HClO_4_ (Suprapur^®^ grade, Carl Roth, Karlsruhe, Germany and 15 ml/100 ml 69% HNO_3_ (Ultrapur^®^ grade, Carl Roth, Karlsruhe, Germany) was added to the plant material following the protocol of Zhao et al. (1994) with the modifications described in Andresen et al. (2020).

### Statistical Analysis

Comparison of groups for finding statistically significant differences compared to Zn treatment and virus infection was performed via the Mann–Whitney U test method in Origin professional (version 2015, Originlab, United States). This test was chosen considering the size of the samples and the fact that most of the photosynthetic parameters do not have normal distribution but become saturated.

## Results

TYMV is readily transmitted mechanically, and PCR analysis confirmed that viral RNA was present in all infected plants (data not shown). After 3 months of treatment, infected plants had typical symptoms such as chlorotic mosaic patches. The plants had a considerable degree of heterogeneity in response to metal treatments. As a result, the fresh shoot biomass of TYMV infected plants was not significantly different from the non-infected plants under the same metal treatment at the *p* < 0.05 level (Supplementary Figure S1). However, growth inhibition by TYMV was observed as the average shoot FW decreased to about 60%, 75% and 50% in infected plants under Zn’0’, Zn10, and Zn100 treatments, respectively, compared to non-infected ones. In Cd-treated plants, shoot growth was inhibited by TYMV by 80%. As expected, shoot biomass of non-infected plants was the highest under optimal Zn availability (Zn10), followed by about 40% lower biomass in the Zn’0’ treatment. In comparison, significant growth inhibition was observed in Zn100 (90%) and Cd treated plants (95%) compared to Zn10.

### Effects of TYMV Infection on Photosynthetic Parameters in *N. ochroleucum*

#### OJIP Chlorophyll Fluorescence Kinetics

OJIP kinetics was measured in a direct imaging way, and tissue heterogeneity of the maximum photochemical quantum yield of PSII reaction centers (Φ_ Po_) and quantum yield of electron transport flux from Q_ A_ to Q_*B*_ (Φ_ ET__2__*o*_) was obtained (Supplementary Figure S2). The visible patchy spread of TYMV caused substantial changes in performance between neighboring cells of Zn’0’, Zn10 and Zn100 leaves. Cadmium treatment itself caused a stronger decrease of photosynthetic performance near the veins compared to the mesophyll away from the veins, and the virus introduced no additional heterogeneity.

To provide a general view of the shapes of the OJIP curves, the data were normalized to F_ p_ (Supplementary Figures S3A–D).

**FIGURE 1:**
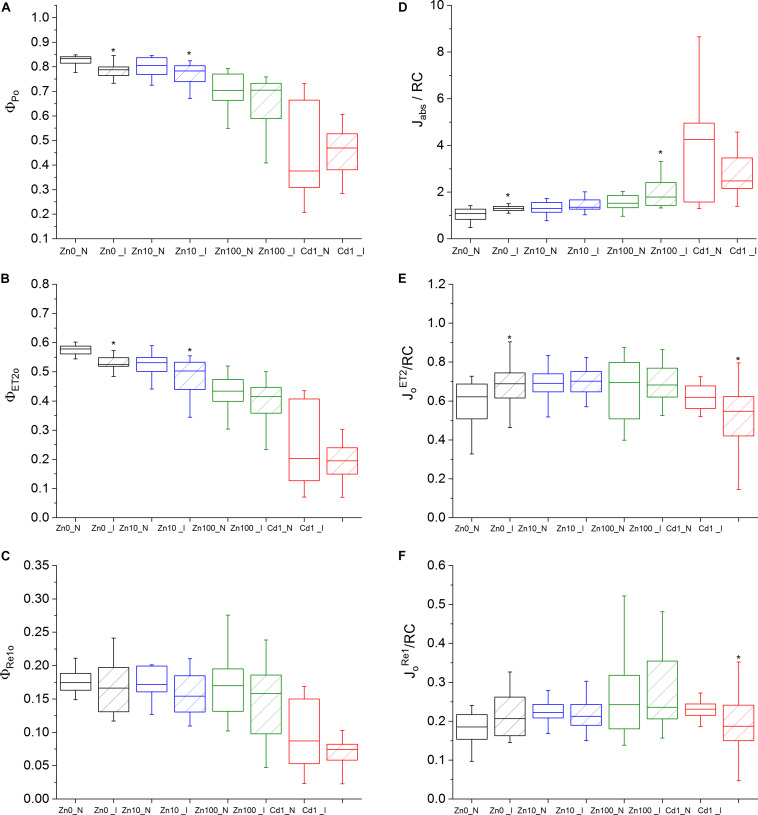
OJIP parameters. Zn0, Zn10, and Zn100 refer to Zn concentrations used in the treatments (‘0’ = 0.01, 10 and 100 μM Zn), N-non-infected – empty box plots. I-infected- box plots with stripes. Cd1 refers to a 1 μM Cd + 10 μM Zn treatment. The line presents the median value (*n* = 6–8), the box shows the values between the 0.25–0.75 percentiles, and the bars show whiskers with 1.5 coefficient for outliers. Asterisks denote significant differences between infected and non-infected *N. ochroleucum* plants within the same Zn treatment according to the Mann–Whitney test. **(A)** Φ_ Po_, maximum quantum yield of primary PSII photochemistry, **(B)** Φ_ ET__2__*o*_, quantum yield of electron transport flux from Q_ A_ to Q_*B*_; **(C)** Φ_*RE*__1__*o*_, quantum yield of electron transport flux until PSI acceptors; **(D)** J^*abs*^/RC, average absorbed photon flux per PSII reaction center, **(E)** J_*o*_^*ET*2^/RC, electron transport flux from Q_ A_ to Q_*B*_ per PSII; **(F)** J_*o*_^*Re*1*o*^/RC, electron transport flux until PSI acceptors according to Stirbet and Govindjee (2011).

Activity parameters were calculated from the OJIP curves to investigate the changes both in response to Zn and Cd treatments, and to TYMV infection. Under Zn’0’ treatment, energy fluxes per reaction center (J_ Abs_/RC, J_*o*_^*ET*2^/RC, J_*o*_^*TR*^/RC and J_*o*_^*Re*1^/RC) significantly decreased compared to Zn10 in non-infected leaves (Supplementary Table S3). Zn and Cd toxicity decreased Φ_ Po_ and Φ_ ET__2__*o*_ and increased J_ Abs_/RC. Additionally, Cd toxicity specifically inhibited the quantum yield of electron transport flux to PSI acceptors (Φ_*Re*__1__*o*_) (Figure 1C). Compared to non-infected Zn10, significant differences in the number of Q_ A_ reducing RCs per PSII antenna Chl (RC/J_ Abs_) were determined in all other metal treatments (Supplementary Figure S4; Supplementary Table S3).

Virus infection in Zn’0’ and Zn10 leaves decreased Φ_ ET__2__*o*_ as well as Φ_ Po_ (Figures 1A,B, significant differences are shown in Supplementary Table S4). In Zn’0’ and Zn100 infected leaves, the maximum trapped exciton flux per PSII (J_*o*_^*TR*^/RC) increased compared to non-infected ones, as well as the average absorbed photon flux per PSII reaction center (or also, apparent antenna size of an active PSII) (J_ Abs_/RC) (Figure 1D and Supplementary Figures S4D,E). In Cd treatment, both the electron transport flux from Q_ A_ to Q_*B*_ per PSII (J_*o*_^*Et*2^/RC) and electron transport flux to PSI acceptors per PSII (J_*o*_^*Re*1^/RC) decreased in infected compared to non-infected leaves (Figures 1E,F).

#### Classical Fluorescence Quenching Analysis

##### Effect of metals treatments

**FIGURE 2:**
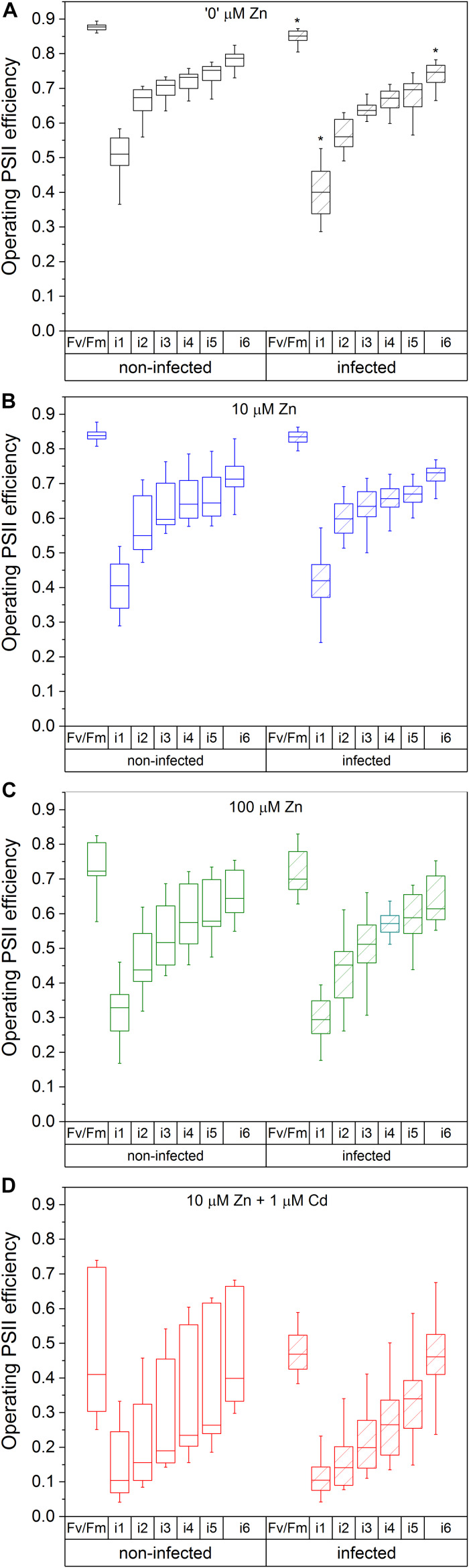
Operating PSII efficiency in the irradiation phase, with the dark-adapted maximal quantum yield of PSIRC (F_*v*_/F_ m_) as a reference. Zn0 **(A)**, Zn10 **(B)**, and Zn100 **(C)** refer to Zn concentrations used in the treatments (‘0’ = 0.01, 10 and 100 μM Zn), Cd1 **(D)** refers to a 1 μM Cd + 10 μM Zn treatment. The line presents the median value (*n* = 6–8), the box shows the values between the (0.25–0.75 percentiles, and the bars show whiskers with 1.5 coefficient for outliers. Asterisks denote significant differences between infected and non-infected *N. ochroleucum* plants within the same Zn treatment according to the Mann–Whitney test. F_ m_, maximal chlorophyll fluorescence in dark-adapted state; F_ m_’, maximal chlorophyll fluorescence in light-adapted state; F_0_, minimal chlorophyll fluorescence in dark-adapted state; F_*v*_, variable fluorescence = (F_ m_-F_0_)/F_ m_; Φ_ PSII_ = (F_ m_’-F_*t*_’)/F_ m_’) from the beginning (i1) to the end (i6) of the 200 s actinic light phase using 600 ms flash of saturating light.)

**FIGURE 3:**
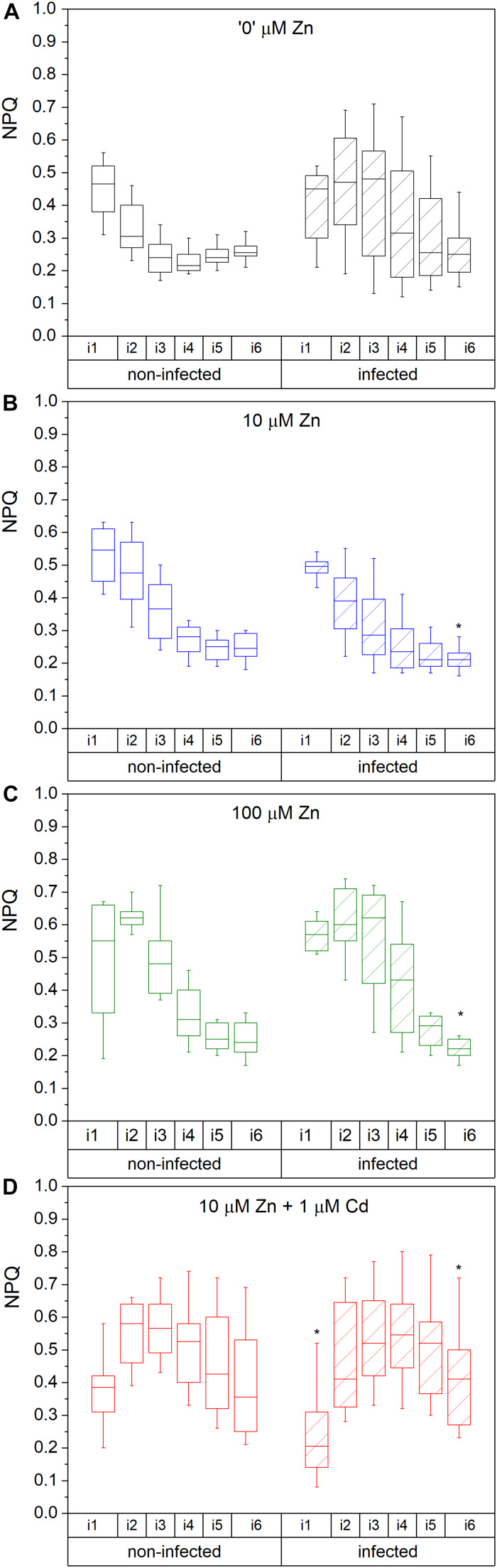
Complete NPQ ((F_ m_-F_ m_’)/F_ m_) during the irradiance phase. Zn0 **(A)**, Zn10 **(B)** and Zn100 **(C)** refer to Zn concentrations used in the treatments (‘0’ = 0.01, 10 and 100 μM Zn), Cd1 **(D)** refers to a 1 μM Cd + 10 μM Zn treatment. The line presents the median value (*n* = 6–8), the box shows the values between the 0.25–0.75 percentiles, and the bars show whiskers with 1.5 coefficient for outliers. Asterisks denote significant differences between infected and non-infected *N. ochroleucum* plants within the same Zn treatment according to the Mann–Whitney test. F_ m_, maximal chlorophyll fluorescence in dark-adapted state; F_ m_’, maximal chlorophyll fluorescence in light-adapted state; NPQ was measured from the beginning (i1) to the end (i6) of the 200 s actinic light phase using 600 ms flashes of saturating light.

The maximal photochemical efficiency of PSII in the dark adapted state (F_*v*_/F_ m_) in non-infected leaves was highest in Zn’0’ treatment followed by Zn10. At the same time, it significantly decreased in the Zn100 and Cd treatments (Figures 2A–D). Similarly, operating PSII efficiency during light acclimation (indicative of electron flow through PSII, Φ_ PSII_) was constantly lower in Zn100 and Cd compared to Zn’0’ and Zn10 treatments in non-infected leaves (Figures 2A–D and Supplementary Table S3). According to these parameters, Zn deficiency did not affect PSII photochemistry, while Zn100 and Cd exposure inhibited PSII efficiency in *N. ochroleucum* (also visible in microscopic images, Supplementary Figure S5). In non-infected leaves, Zn’0’ and Zn100 treatments increased the relaxation of PSII operating efficiency compared to Zn10, while Cd treatment significantly reduced this parameter (Supplementary Figure S6 and Supplementary Table S3). Complete non-photochemical quenching, qCN (here named NPQ) = (F_ m_-F_ m_’)/F_ m_, during illumination phase gradually decreased in Zn’0’ and Zn10 treatments from NPQ_i1 to NPQ_i6, while in Zn100 it remained at higher level for a longer period before starting to decrease (Figures 3A–C). In response to Cd treatment, NPQ had very different dynamics (hyperbolic shape) (Figure 3D). NPQ in the dark relaxation phase (Supplementary Figure S7) slowly decreased from NPQ_r1 to NPQ_r5, indicating the recovery of photosynthetic components in all metal treatments. The highest NPQ_r1 was observed in Cd treated leaves.

##### Modifications induced by virus infection

Virus infection induced a significant decrease in F_*v*_/F_ m_ and Φ_ PSII_ at i_1 and i_6 phase in Zn’0’ (Figure 2A, significant difference shown for the 1st and the 6th phase, *p* < 0.001, Supplementary Table S4 and Supplementary Figure S5). In contrast, no significant effect of virus infection was observed in other treatments. Relaxation of Φ_ PSII_ toward reaching F_*v*_/F_ m_ after the actinic light phase was decreased by TYMV infection in Zn’0’. In the other treatments there was no significant effect of the virus (Supplementary Figure S6 and Supplementary Table S4), although lower median values were obtained in Zn10 infected leaves through the whole period of dark relaxation. Under our experimental conditions, Φ_ PSII__r5 was not able to recover in infected leaves to F_*v*_/F_ m_ values in Zn10 and Zn100 treatments, contrary to Zn’0’ and Cd treatments. In Zn’0’ and Zn100 infected leaves, the decrease of NPQ in the irradiance phase was slower than in non-infected ones (Figures 3A–C). In infected Cd-treated leaves, NPQ_i1 was reduced by 50% compared to non-infected ones, and NPQ values remained higher through the entire irradiance phase (Figure 3D and Supplementary Table S4). Virus infection significantly reduced NPQ_r1 and NPQ_r5 in Zn’0’ and Zn10 treatments, while no effects were observed in Zn100 and Cd-treated leaves (Supplementary Figure S7 and Supplementary Table S4).

In summary, the analyses of photosynthetic parameters indicated higher sensitivity of PSII to TYMV infection in Zn-deficient and toxic Zn treatments, compared to optimal Zn supply, whereas photosynthetic inhibition by Cd itself was much stronger even in non-infected leaves. Therefore, the changes in the leaf Zn/Cd concentration and distribution in response to the virus were investigated further.

### Transcription Regulation of ATPases (*HMA3*, *HMA4*), Cation Diffusion Facilitator *MTP1* and ZIP Transporter *ZNT5* From *N. ochroleucum* (Organ and Cellular Level) Analyzed by RT – Real Time PCR and QISH

**FIGURE 4:**
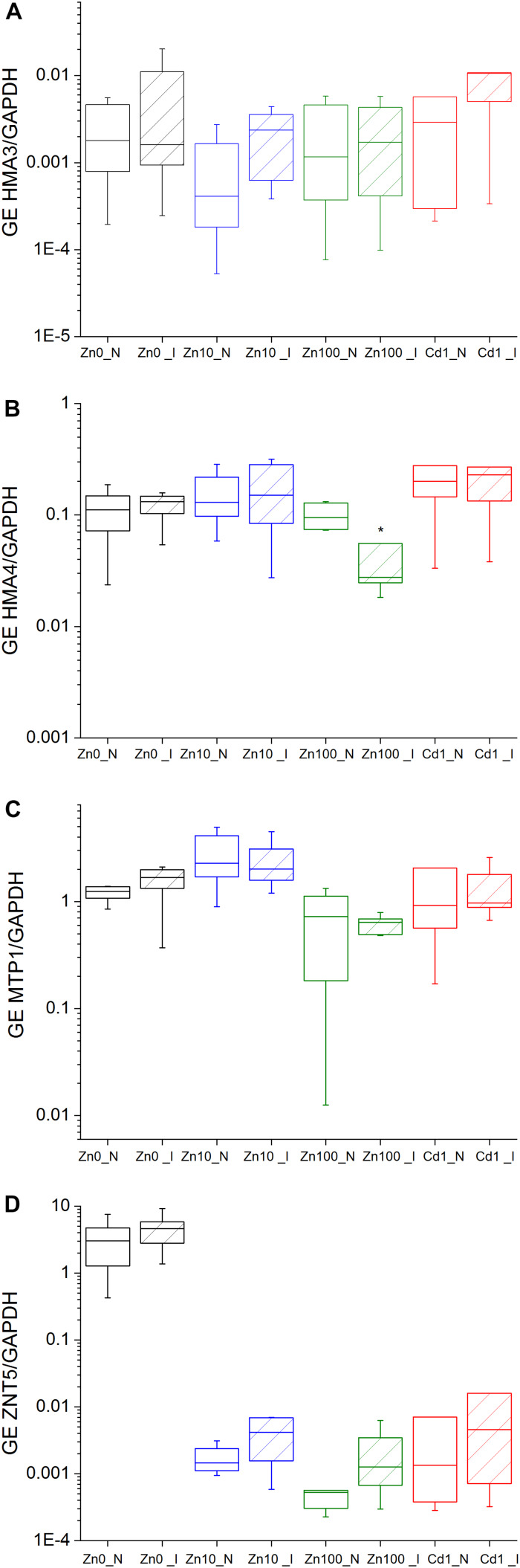
Expression of metal transporter genes normalized to cytosolic *GAPDH* in the leaves of *N. ochroleucum* plants calculated according to Pfaffl. Zn0 **(A)**, Zn10 **(B)** and Zn100 **(C)** refer to Zn concentrations used in the treatments (‘0’ = 0.01, 10 and 100 μM Zn), Cd1 **(D)** refers to a 1 μM Cd + 10 μM Zn treatment. N – non-infected – empty box plots. I – infected – box plots with stripes. The line presents the median value (*n* = 6–8), the box shows the values between the 0.25–0.75 percentiles, and the bars show whiskers with 1.5 coefficient for outliers. Asterisks denote significant differences between infected and non-infected plants within the same Zn treatment according to the Mann–Whitney test.

Gene expression (GE) of several metal transporters was analyzed on the transcript level to determine the changes in Zn distribution

under TYMV infection, both using total mRNA normalized to *GAPDH* and *18S*, and mRNA *in situ* hybridization normalized to *GAPDH*. Significantly upregulated GE of *ZNT5*/*18S* and *ZNT5*/*GAPDH* and *HMA3*/*18S* was observed in response to Zn deficiency (Zn’0’) compared to Zn10 and Zn100 treatments (Supplementary Table S5). Virus infection slightly upregulated GE of *ZNT5*/*GAPDH* compared to respective controls and increased GE of *HMA3*/*GAPDH* in Zn10 infected leaves (Figures 4A–D and Supplementary Table S6). In infected Zn100 leaves, GE of *HMA4*/*GAPDH* was significantly downregulated.

**FIGURE 5:**
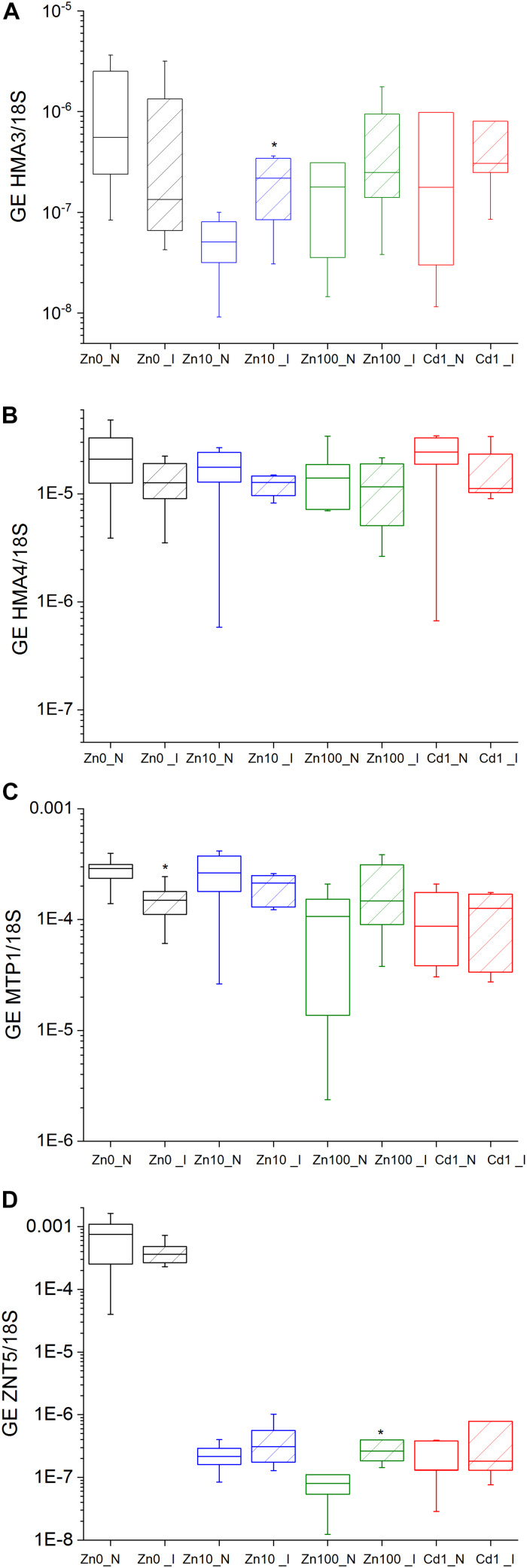
Expression of metal transporter genes normalized to *18S* in the leaves of *N. ochroleucum* plants calculated according to Pfaffl. Zn0 **(A)**, Zn10 **(B)** and Zn100 **(C)** refer to Zn concentrations used in the treatments (‘0’ = 0.01, 10 and 100 μM Zn), Cd1 **(D)** refers to 1 μM Cd + 10 μM Zn treatment. N – non-infected – empty box plots. I – infected – box plots with stripes. The line presents the median value (*n* = 6–8), the box shows the values between the 0.25–0.75 percentiles, and the bars show whiskers with 1.5 coefficient for outliers. Asterisks denote significant differences between infected and non-infected plants within the same Zn treatment according to the Mann–Whitney test.

Normalization to *18S*-rRNA as an indicator of overall transcription activity (Küpper et al., 2007b) revealed significantly upregulated GE of *HMA3*/*18S* in the leaves of Zn10 infected leaves (Figures 5A–D and Supplementary Table S6). Significantly upregulated GE of *ZNT5* was observed in infected Zn100 leaves, followed by a slight increase in GE of *MTP1*/*18S* and *HMA3*/*18S*. In Zn’0’ infected leaves, *HMA3* and *MTP1* GE were downregulated, while no significant differences in relation to TYMV were observed in Cd treatments.

**FIGURE 6:**
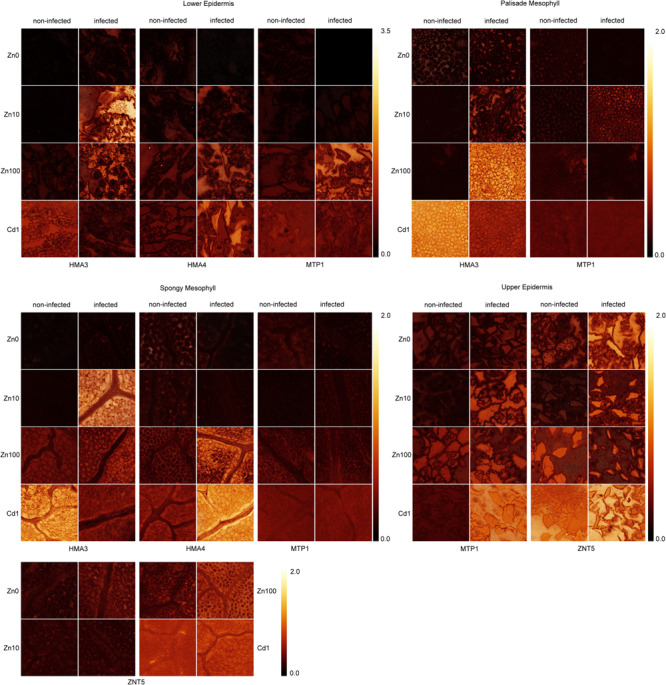
Images showing the gene expression ratio in different tissues of metal transporters to cGADPH probe fluorescent signals of TYMV infected and non-infected *N. ochroleucum* leaves. Zn0, Zn10, and Zn100 refer to Zn concentrations used in the treatments (‘0’ = 0.01, 10 and 100 μM Zn), Cd1 refers to a 1 μM Cd + 10 μM Zn treatment. The scale bars for all genes within the same tissue are shown.

QISH analysis showed that *MTP1* was mostly expressed in epidermal storage cells, followed by bundle sheath cells and spongy mesophyll cells (Figure 6). Virus infection slightly increased *MTP1* GE in the upper epidermis, palisade and spongy mesophyll and bundle sheath cells of Zn10 leaves, and in the lower epidermis of infected Zn100 leaves. In Cd treated infected leaves, *MTP1* GE in the upper epidermis was higher than in non-infected ones (Figure 6).

In response to the virus, *ZNT5* GE increased in the upper epidermis and spongy mesophyll of Zn’0’ and Zn10 leaves and in bundle sheath cells in Zn100 leaves compared to non-infected ones.

*HMA3* GE was increased by infection in the epidermis of all Zn treated plants, in the spongy mesophyll of Zn10 leaves, and in palisade mesophyll of Zn100 infected leaves compared to non-infected ones.

TYMV infection decreased *HMA4* GE in the lower and upper epidermis of Zn’0’ leaves, oppositely to Cd and Zn100 treatments where its abundance increased in the lower epidermis, and spongy mesophyll compared to non-infected leaves. In Zn10 treatment, high variability in the *HMA4* GE was observed, and no clear trend in response to infection could be concluded. In the palisade mesophyll the results were noisy across all treatments (Figure 6). As a negative control, the signal intensity of non-binding oligonucleotide normalized to GADPH in the same way as genes of interest is shown in Supplementary Figure S9.

In summary, according to the QISH results, the most prominent changes in GE of metal transporter genes in response to TYMV were in Zn deficient leaves in which upregulation of *ZNT5* and downregulation of *HMA4* were observed. On the other hand, TYMV infection increased GE of *HMA3* (mesophyll) and *MTP1* (epidermis) in Zn10 and Zn100 leaves, indicating a redistribution of Zn in response to the virus.

### Metal Accumulation in *N. ochroleucum* Leaves

Accumulation of the essential metals, Fe, Zn, Cu and Ni in leaves of non-infected and infected plants was heterogeneous. Nevertheless, Zn content expectedly increased with increased Zn concentration in the nutrient solution (Supplementary Figure S8 and Supplementary Tables S7, S8). In infected Zn’0’ leaves, median Zn and Ni contents were higher (51 mg.kg^–1^ Zn, 77 mg.kg^–1^ Ni) than in non-infected ones (24 mg.kg^–1^ Zn, 37 mg.kg^–1^ Ni). Slightly higher Fe content was determined in the leaves of Zn’0’ and Zn10 infected leaves compared to non-infected ones. In Zn100 infected leaves, median Zn content increased by about 40%, and Fe content decreased. Cu concentration was not significantly altered by different Zn treatments and infection, while a significant accumulation was observed in Cd treated leaves compared to Zn10 (Supplementary Table S8). Slightly increased Zn concentration in Zn deficient and Zn100 infected plants may indicate its involvement in plant defense responses.

### MicroXRF and Tomography Analysis of Zn Cellular Distribution

**FIGURE 7:**
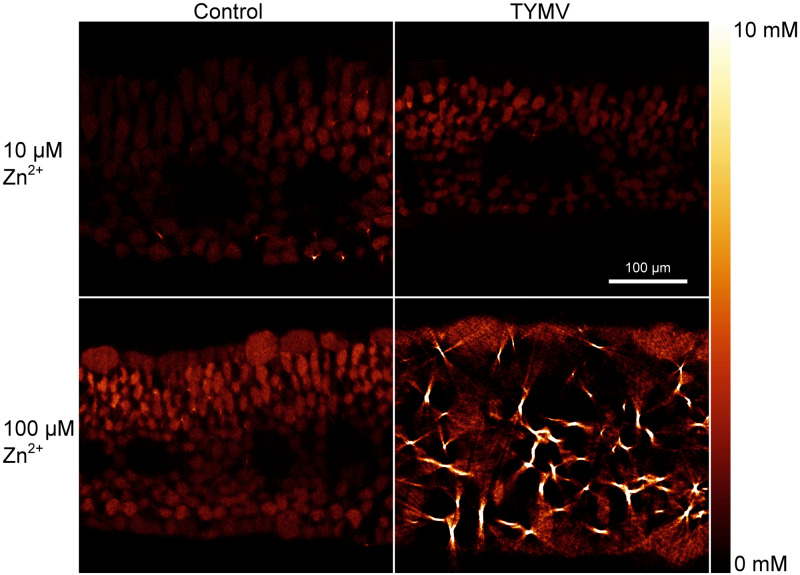
μXRF tomography of Zn distribution in fully developed *N. ochroleucum* leaves. The samples were taken at the time of harvest of the plants. At least two replicates from independent experiments were measured for each panel of this figure; representative examples are shown. **Top:** plants grown with 10 μM Zn^2+^; **bottom:** 100 μM Zn^2+^; Left: non-infected plants; right: TYMV-infected plants. The scale bar in the top-right map is valid for all four maps.

Zinc distribution on the cellular level was compared between infected and non-infected leaves in Zn10 and Zn100 treatments. Zinc was mainly localized in the vacuoles of the mesophyll, to a higher extent than in the epidermis, and it was not visible in the veins (Figure 7). This is in contrast to the closely related model hyperaccumulator *N. caerulescens* (Küpper et al., 1999; Leitenmaier and Küpper, 2011), and most other hyperaccumulator species (review: Leitenmaier and Küpper, 2013) which preferentially sequester hyperaccumulated metals in the epidermis. In the Zn100 treatment, more Zn accumulated in the epidermis than in the Zn10 treatment, while the mesophyll concentrations did not differ much, and in the epidermis, the highest Zn was found in the vacuoles of the largest cells like in *N. caerulescens* (Küpper et al., 1999; Leitenmaier and Küpper, 2011). Furthermore, we observed Zn-biomineralization dots in the mesophyll cell walls of Zn100 leaves, which were more pronounced in the infected than in the non-infected tissues (Figure 7). These results correlate with the observed changes in GE determined by QISH, indicating mobilization of Zn in Zn-sufficient and excessive Zn treatments.

## Discussion

Under our experimental conditions, *N. ochroleucum* can be regarded as Zn tolerant, since in the 10 μM Zn treatment it had higher growth compared to 0.01 μM Zn. This concentration is sublethal for more sensitive species such as crops, which may suffer from complete growth arrest under the same conditions. Furthermore, although the shoot biomass significantly decreased in the 100 μM Zn and 1 μM Cd treatment, the plants were able to partially acclimatize to excess metals. This was evident by photosynthetic activity and similar GE of *GAPDH* and *18S* compared to the 10 μM Zn treatment.

Long-term exposure to TYMV in this study was used to ensure systemic infection, and although the accumulation of the virus was not quantified, all infected plants showed TYMV infection symptoms (also confirmed by PCR amplification of the virus cDNA), which allowed us to investigate interactions between effects of Zn and Cd treatments and the virus.

### Virus Effects on Chlorophyll Fluorescence Kinetics in Relation to Zn Availability and Cd Toxicity

The most striking TYMV-induced inhibition of photosynthetic light reactions was observed in Zn-deficient, Zn’0’, plants. Increased minimal chlorophyll fluorescence in dark-adapted state, F_*o*_ (visible in the OJIP induction curve), indicated damage to antenna complexes and reduced energy interaction between the antenna and PSIIRC. This was supported by the increased number of absorbed photons per PSII RC (J_ Abs_/RC), reduction of the maximal quantum yield of PSII (Φ_ Po_ and F_*v*_/F_ m_), and the efficiency of electron transport between Q_ A_ and Q_*B*_ (Φ_ ET__2__*o*_). Such inactivation of RCs is proposed to protect the leaf against photo-oxidative damage under excess light. However, under biotic stress, this may be a consequence of damaged chloroplast ultrastructure, especially thylakoids, by the virus infection (Dreher, 2004; Li et al., 2016). Under Zn10 conditions, TYMV decreased Φ_ Po_ and Φ_ ET__2__*o*_ as well, but in Zn100, these parameters were not affected by the virus. This might indicate that the high Zn diminished the virus effects, but more likely it means that disturbance of PSII efficiency by Zn toxicity (also evidenced by severe growth arrest) was more pronounced than the virus effect. In this context, it is particularly interesting that the virus-induced inhibition of Φ_ Po_ and Φ_ ET__2__*o*_ exhibited pronounced patchiness in all metal treatments except Cd, where Cd toxicity seemed to dominate over the virus effects. Cd induced a significant increase of F_*o*_ regardless of infection, while a decrease in fluorescence slope at the J, I, and P steps indicated impaired ET on both the donor and the acceptor side of PSII. Correspondingly, operating PSII efficiency was impaired by Cd toxicity, in line with an earlier study on the related *N. caerulescens* (Küpper et al., 2007a) where the parameters of the fast kinetics could not yet be measured. The sensitivity of PSI to TYMV was observed as a decreasing trend in quantum yield of electron transport toward PSI acceptors in Zn treatments, while again Cd toxicity itself induced disturbed electron flow to PSI that masked the virus effect.

Classical fluorescence quenching analysis further revealed TYMV inhibition of operating PSII efficiency in the Zn’0’ treatment. Furthermore, the decrease of non-photochemical quenching during light adaptation was slower in Zn’0’ and Zn100 infected leaves, and generally less in Cd treated plants, compared to non-infected leaves. Immediately upon illumination of a dark-adapted leaf, NPQ usually increases due to initiation of electron transport and ΔpH formation, which precede the activation of ATP synthase. Afterward, NPQ decreases with activation of the Calvin-Benson cycle (Baker, 2008). Continuous and severe stress, however, can affect NPQ regulation in various ways. The delayed and diminished NPQ regulation might be due to the loss of RCs since less variable fluorescence is produced, or due to decreased linear electron transport rate and a smaller *trans-*thylakoid proton gradient, as well as due to lipid peroxidation and membrane damage caused by the virus.

The described effects on photochemistry and non-photochemical quenching can be explained by the general fact that TYMV infections are predominately targeted to the chloroplasts, inducing cytopathological changes in their structure, chlorophyll degradation and disturbance in photosynthesis (Guo et al., 2005; Zhao et al., 2016). Downregulation of transcript levels of the photosynthetic light reactions, carbon reduction cycle and pigment synthesis genes has been observed as well (Yang et al., 2007; Alexander and Cilia, 2016; Ni et al., 2017).

As a redox inert, essential micronutrient, Zn^2+^ is a cofactor of many enzymes, including those involved in DNA transcription, protein, lipid, nucleic acid and carbohydrate metabolism (Broadley et al., 2007; Ishimaru et al., 2011; Marschner, 2012). It is also a structural component in proteins with Zn-finger domains, which make about 22% of transcription factors in *A. thaliana* (Riechmann et al., 2000), emphasizing the role of Zn in regulation of gene expression. Zn deficiency may inhibit photosynthesis due to altered biosynthesis and function of photosynthetic components. Activity of Cu/Zn-SOD may be inhibited under Zn deficient conditions. This leads to accumulation of superoxide anion radical in the chloroplasts, causing oxidative stress. Besides, a role of chloroplastic carbonic anhydrase (with Zn^2+^ in the active site) in hypersensitive response to virus infection through salicylic acid binding has been proposed (Slaymaker et al., 2002). Another recently discovered zinc finger protein, LOW QUANTUM YIELD OF PSII 1 (LQY1) localized in the thylakoid membrane, seems to be responsible for PSII repair and assembly of photodamaged PSII complexes (Lu et al., 2011). Zn deficiency-induced breakdown of carbon metabolism increased the content of starch and sucrose, while a decrease in RNA and protein synthesis was observed, as well as decreased activity of FBP aldolase in the glycolysis pathway (Suzuki et al., 2012).

Excess Zn may induce oxidative stress through inhibition of antioxidative enzymes, induction of Fe deficiency, and by stabilizing redox-active quinhydrones in the cell wall (Cuypers et al., 2002; Morina et al., 2010, 2016). In chloroplasts, direct effects of Zn toxicity may be attributed to the replacement of other metal ions from active sites, such as Mg in chlorophylls resulting in reduced charge separation and increasing heat dissipation (Küpper et al., 2002; Paunov et al., 2018), as well as inhibition of RuBP carboxylase capacity (Van Assche and Clijsters, 1986).

In this experiment, in deficient (Zn’0’) and in some cases in Zn10 treatments, virus effects on PSII were enhanced compared to the toxic Zn100 and Cd treatments, which already induced severe disturbances of PSII photochemistry and so that the virus effects became masked or visible only in relation to PSI. In addition, shoot growth was less affected by the virus in Zn10 than in Zn’0’ plants. These results already show that optimal Zn supply can support plant vigor even under virus infection.

### Metal Distribution and Expression of Metal Transporter Genes and Interactions With TYMV

In this study, total metal content in the shoots and whole-organ GE of *MTP1*, *ZNT5*, *HMA3* and *HMA4* have been correlated with tissue and cellular distribution of Zn and tissue GE of the same metal transporters, in response to TYMV. The results emphasized the importance of investigating the virus-induced effects on metal homeostasis on both the whole-tissue and the cellular level. While the whole-organ data obtained with ICP-MS for the metals and qPCR for the GE have technically a higher precision and show the best whole-organ average, they do not reflect the fine changes on the cellular level, where local increases and decreases may cancel out each other in the average. Therefore, the spatially resolved (imaging) measurements of the single-cell level by μXRF (metals) and QISH (mRNA) were important.

Significantly increased *MTP1* GE in Zn10 compared to Zn’0’ plants may indicate that Zn tolerance in this species is achieved by Zn sequestration to the vacuole, while under excessive Zn (100 μM) GE of *HMA3*/*18S* was more pronounced compared to Zn10. This correlated with the data from μXRF, showing that in the Zn100 treatment surplus Zn became stored in the epidermal vacuoles, while in Zn10 the metabolically needed Zn was allocated mostly to the mesophyll. In contrast, in hyperaccumulator plants, some of the metal transporter genes such as *HMA4* are naturally overexpressed in comparison to non-hyperaccumulators (Leitenmaier and Küpper, 2013). In a recent study (Mishra et al., 2017), distinct patterns in the mRNA levels of *HMA4* reflected the main storage sites in the two hyperaccumulator species, epidermal cells in *N. caerulescens* and mesophyll cells in *A. halleri*.

In infected Zn-deficient *N. ochroleucum*, upregulation of *ZNT5* GE in spongy mesophyll and upper epidermis, and downregulation of apoplastic loading by *HMA4*, could indicate enhanced cellular loading of Zn in the presence of TYMV. This may help to support photosynthetic activity to a certain extent. Enhanced Ni accumulation in response to Zn deficiency was observed in *A. thaliana* roots; however, it was unclear which transporter was involved in Ni uptake (Nishida et al., 2015). Further, it is unknown if and in how far Ni is involved in response to viral infection under Zn deficiency as both metals are chemically very different and normally have no overlap of enzymatic functions (Andresen et al., 2018). One possibility is through increased urease activity and modifications of N metabolism.

Contrastingly to Zn’0’, strongly enhanced vacuolar Zn sequestration (through activation of *HMA3* and *MTP1*) was noticed in Zn10 and Zn100 infected leaves. In addition, *ZNT5* GE increased in the epidermis of Zn10 and bundle sheath cells of Zn100 plants, indicating increased cellular loading of Zn. Enhanced *HMA3* GE in the mesophyll of both Zn10 (spongy) and Zn100 (palisade) infected leaves, may be behind the observed change in (sub)cellular Zn compartmentation. The total leaf metal content did not differ between Zn10 and Zn100 infected and non-infected plants. However, analysis on the cellular level revealed that the virus infection could lead to a local Zn re-distribution from its vacuolar storage to biomineralization dots in the cell walls, correlated with enhanced *HMA4* in Zn100 infected plants. Recently, we used X-Ray Absorption Near Edge Structure (XANES) spectroscopy to investigate the cell-specific changes of Zn ligands in the TYMV-infected leaves. The formation of Zn-biomineralization probably in the form of Zn-silicate, as well as Zn binding to histidine and phosphate, were induced by the virus (Mijovilovich et al., 2019).

In Cd treated infected plants, we observed increased *HMA4* GE in spongy mesophyll and lower epidermis, which together with increased *MTP1* levels in the mesophyll may indicate enhanced Cd sequestration in the vacuoles and the cell wall in infected leaves.

Evolutionary advantage of metal hyperaccumulation as a defense system against mainly herbivores, but also pathogens, has been proposed in Zn/Cd hyperaccumulating species. With this respect, metal accumulation may be accompanied by induction of organic defense, such as accumulation of glucosinolates and/or induction of salicylic acid signaling pathway. Activation of metal transporter genes and Zn mobilization as a part of plant defense responses to virus infection has not been explored, especially in non-hyperaccumulating species. The results presented in this study show that adequate Zn supply is needed for maintaining the plant primary metabolism under prolonged TYMV infection. Sensitivity of PSII and electron transfer chain to TYMV-induced damage was the highest in Zn deficient plants. At the same time, excessive Zn and Cd acted synergistically with TYMV, inducing growth inhibition and overall inhibition of photochemistry. Interactions between Zn metabolism and TYMV infection were evident from preferential Zn accumulation in the mesophyll, activation of vacuolar metal transporters and Zn biomineralization in mesophyll cell walls. The study emphasizes the importance of monitoring metal distribution, photosynthesis and activity of metal transporters on the tissue/cellular level rather than bulk tissue. Further work is needed to reveal the global effects of Zn on plant metabolism under virus infection, particularly of chloroplastic metal transporters.

## Data Availability Statement

The datasets generated for this study are available on request to the corresponding author.

## Author Contributions

HK initiated the work on plant-metal-pathogen interactions in the lab and supervised the project. ArM had the idea of working on plant–virus interactions; the use of TYMV as the model virus was suggested by JŠ. FM did the sample preparation and real-time PCR measurements, analyzed and interpreted the data from all (also FKM) measurements. ArM performed the metal treatments, optimized the real-time PCR conditions and performed FKM and QISH measurements. AnM and HK performed the μXRF measurements, AnM performed the fits with Geopixe, DB did the tomographic reconstructions. ŠM did the ICP-MS measurements. FM performed the statistics. FM and ArM wrote the first draft of the manuscript, later versions were written by FM with revisions by all authors.

## Conflict of Interest

The authors declare that the research was conducted in the absence of any commercial or financial relationships that could be construed as a potential conflict of interest.
