# Supplementary material for: Interaction Between Zn Deficiency, Toxicity and Turnip Yellow Mosaic Virus Infection in Noccaea ochroleucum
Source: Front Plant Sci. 2020 Jun 5;11:739. doi: 10.3389/fpls.2020.00739 (PMC7290001; doi:10.3389/fpls.2020.00739)
Supplement: Supplementary file 1 [file Data_Sheet_1.PDF]

Supplementary Information

Interaction between Zn deficiency, toxicity and Turnip yellow mosaic virus infection in *Noccaea ochroleucum*

Filis Morina, Archana Mishra[1,#], Ana Mijovilovich[1], Šárka Matoušková[2], Dennis Brückner[3,4,5], Josef Špak[6], Hendrik Küpper[1,7,*]

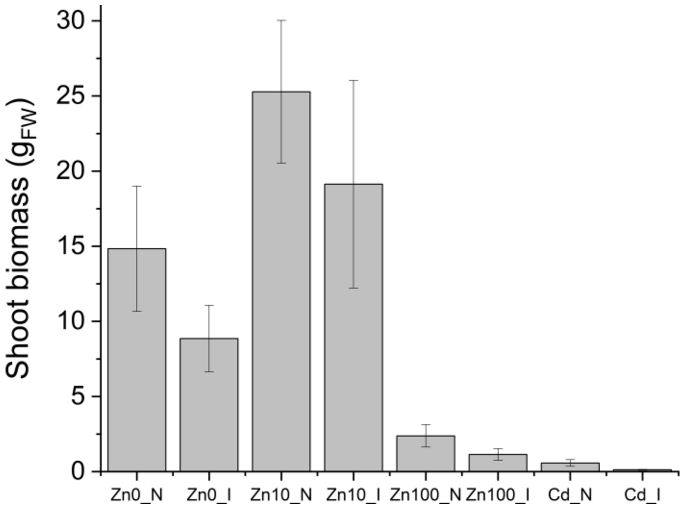

Supplementary Figure S1. Fresh shoot biomass of *N. ochroleucum* shoots after three months of treatment with different Zn/Cd and TYMV infection. Zn0, Zn10 and Zn100 refer to Zn concentrations used in the treatments (‘0’ = 0.01, 10 and 100 µM Zn), Cd1 refers to 1 µM Cd + 10 µM Zn treatment. Values represent averages ± SE (n=4-8). N-non-infected, I-infected.

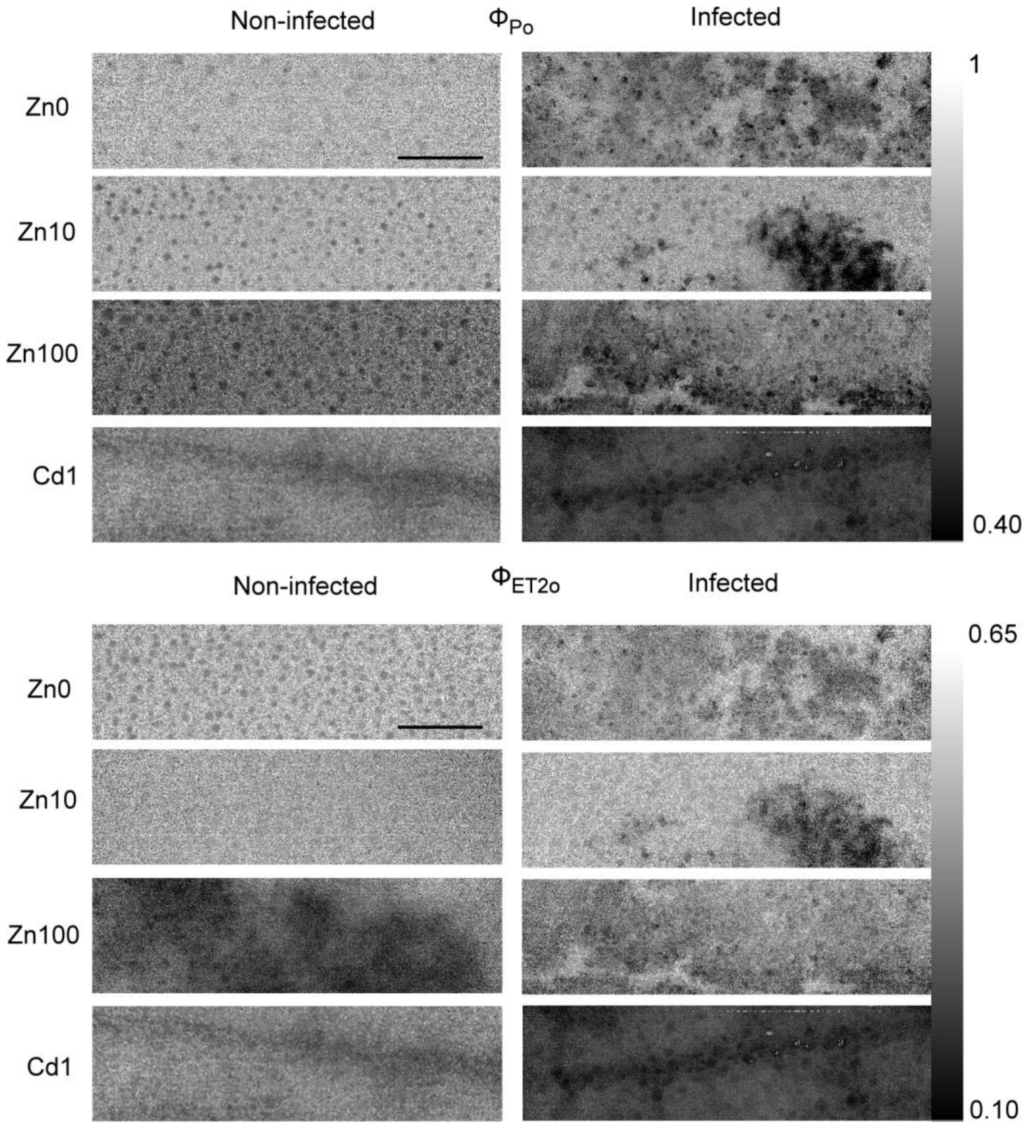

Supplementary Figure S2. Microscopic images of OJIP parameters of *N. ochroleucum* leaves. Zn0, Zn10 and Zn100 refer to Zn concentrations used in the treatments ('0' = 0.01, 10 and 100 µM Zn), Cd1 refers to 1 µM Cd + 10 µM Zn treatment. Left: non-infected plants; right: TYMV-infected plants. Φ_{Po} is a measure of maximum quantum yield of primary PSII photochemistry, and Φ_{ET2o} is a measure for the quantum yield of the electron transport flux from Q_A to Q_B. Scale bar is 300 µm.

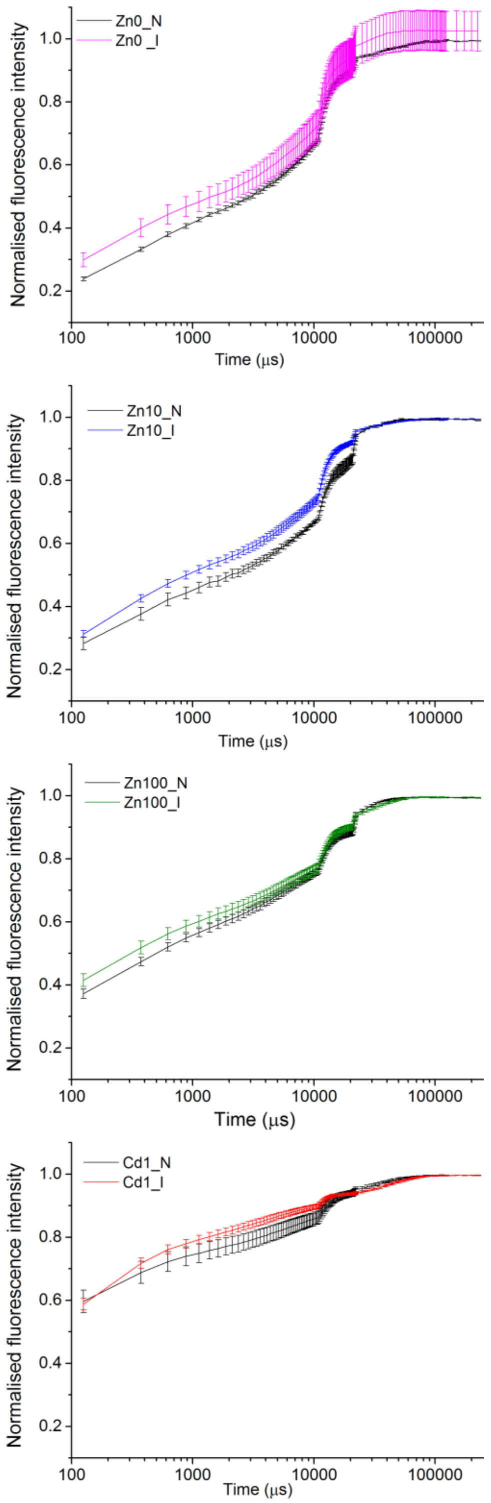

Supplementary Figure S3. OJIP transients of non-infected and infected N. *ochroleucum* leaves. Fluorescence intensity was normalized to F_m (F_P). Zn0, Zn10 and Zn100 refer to Zn concentrations used in the treatments (‘0’ = 0.01, 10 and 100 µM Zn), Cd1 refers to 1 µM Cd + 10 µM Zn treatment. Values present average ± SE (n=25-27).

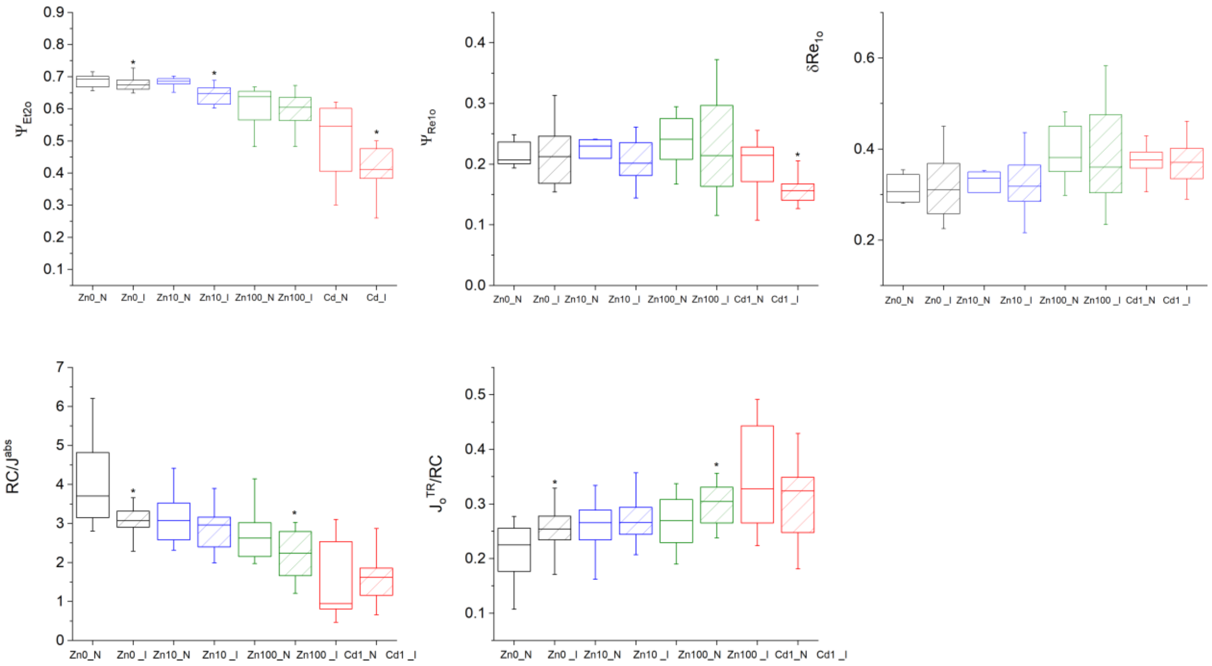

Supplementary Figure S4. OJIP parameters, efficiencies of electron transport and energy fluxes. Zn0, Zn10 and Zn100 refer to Zn concentrations used in the treatments (‘0’ = 0.01, 10 and 100 µM Zn), N-non-infected – empty box plots. I-infected- box plots with stripes. Cd1 refers to 1 µM Cd + 10 µM Zn treatment. The line presents the median value (n=6-8), box shows the values between 0.25-0.75 percentile, and the bars show whiskers with 1.5 coefficient for outliers. Asterisks denotes significant differences between infected and non-infected *N. ochroleucum* plants within the same Zn treatment according to Mann-Whitney test. , Ψ_{ET2o}, Efficiency/probability with which a PSII trapped electron is transferred from Q_A to Q_B; Ψ_{RE1o}, Efficiency/probability with which a PSII trapped electron is transferred until PSI acceptors; δ_{RE1o}, Efficiency/probability with which an electron from Q_B is transferred until PSI acceptors; RC/J^{abs}, Number of Q_A reducing RCs per PSII antenna Chl; J_o^{TR}/RC, Maximum trapped exciton flux per PSII according to Stirbet and Govindjee (2011).

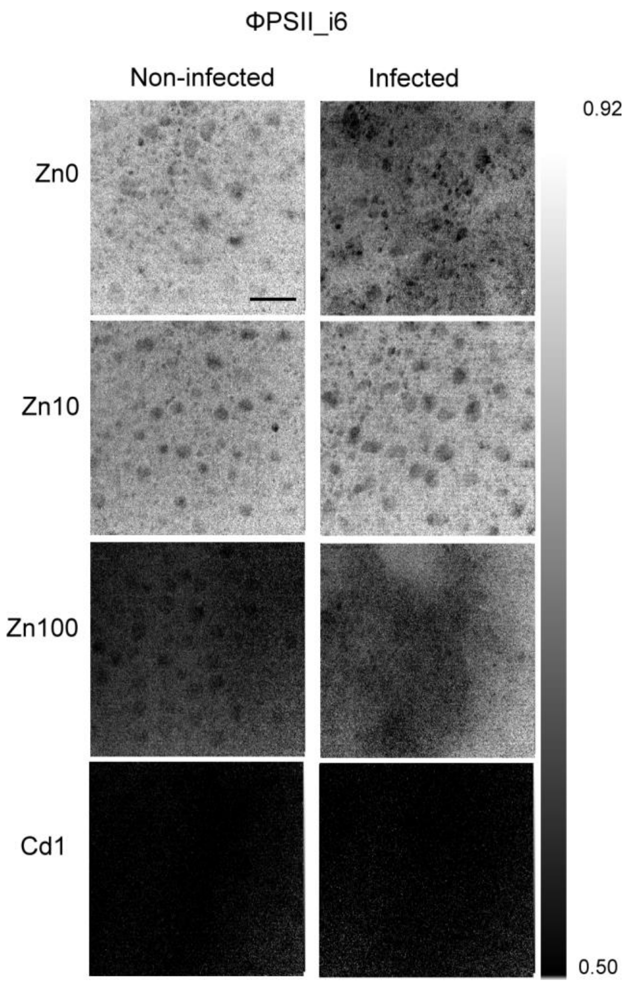

Supplementary Figure S5. Microscopic images of Φ_{PSII_i6} (operating efficiency of PSII after adaptation to actinic light) of *N. ochroleucum* leaves. Zn0, Zn10 and Zn100 refer to Zn concentrations used in the treatments (‘0’ = 0.01, 10 and 100 µM Zn), Cd1 refers to 1 µM Cd + 10 µM Zn treatment. Left: non-infected plants; right: TYMV-infected plants. Scale bar is 300 µm.

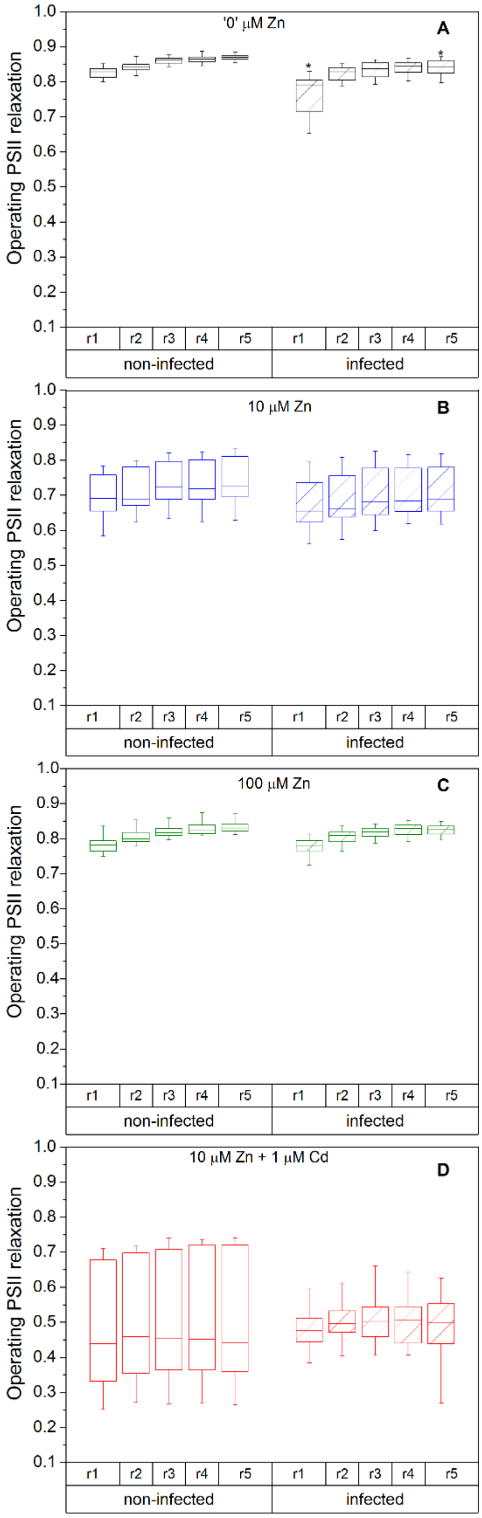

Supplementary Figure S6. Operating PSII efficiency relaxation measured by saturating light flashes during 200 s after switching off the actinic light. Zn0, Zn10 and Zn100 refer to Zn concentrations used in the treatments (‘0’ = 0.01, 10 and 100 μM Zn), Cd1 refers to 1 μM Cd + 10 μM Zn treatment. The line presents the median value (n=6-8), box shows the values between 0.25-0.75 percentile, and the bars show whiskers with 1.5 coefficient for outliers. Asterisks denotes significant differences between infected and non-infected *N. ochroleucum* plants within the same Zn treatment according to Mann-Whitney test. $\Phi_{PSII} = (F_m' - F_t')/F_m'$ from the beginning (r1) to the end (r5) of the 200 s relaxation phase using 600 ms flash of saturating light.

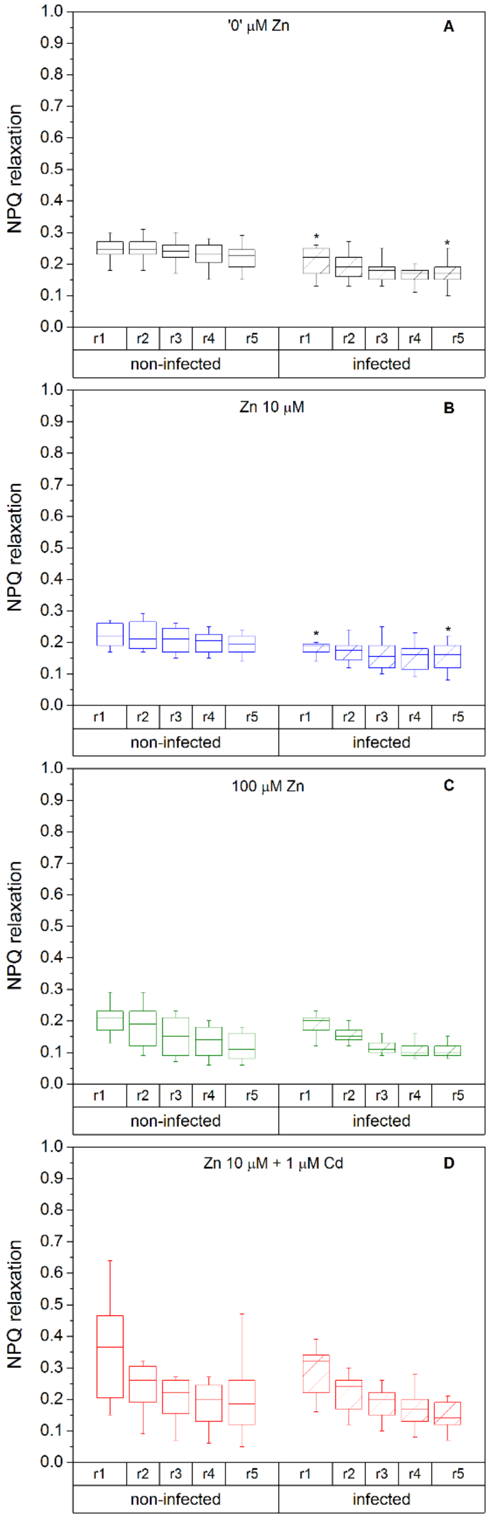

Supplementary Figure S7. Complete NPQ = (F_m-F_m')/F_m relaxation. Zn0, Zn10 and Zn100 refer to Zn concentrations used in the treatments ('0' = 0.01, 10 and 100 µM Zn), Cd1 refers to 1 µM Cd + 10 µM Zn treatment. The line presents the median value (n=6-8), box shows the values between 0.25-0.75 percentile, and the bars show whiskers with 1.5 coefficient for outliers. Asterisks denotes significant differences between infected and non-infected *N. ochroleucum* plants within the same Zn treatment according to Mann-Whitney test. F_m, maximal chlorophyll fluorescence in dark-adapted state; F_m', maximal chlorophyll fluorescence in light-adapted and relaxation state; NPQ was measured from the beginning (r1) and the end (r5) of the 200 s dark relaxation phase using 600 ms flash of saturating light.

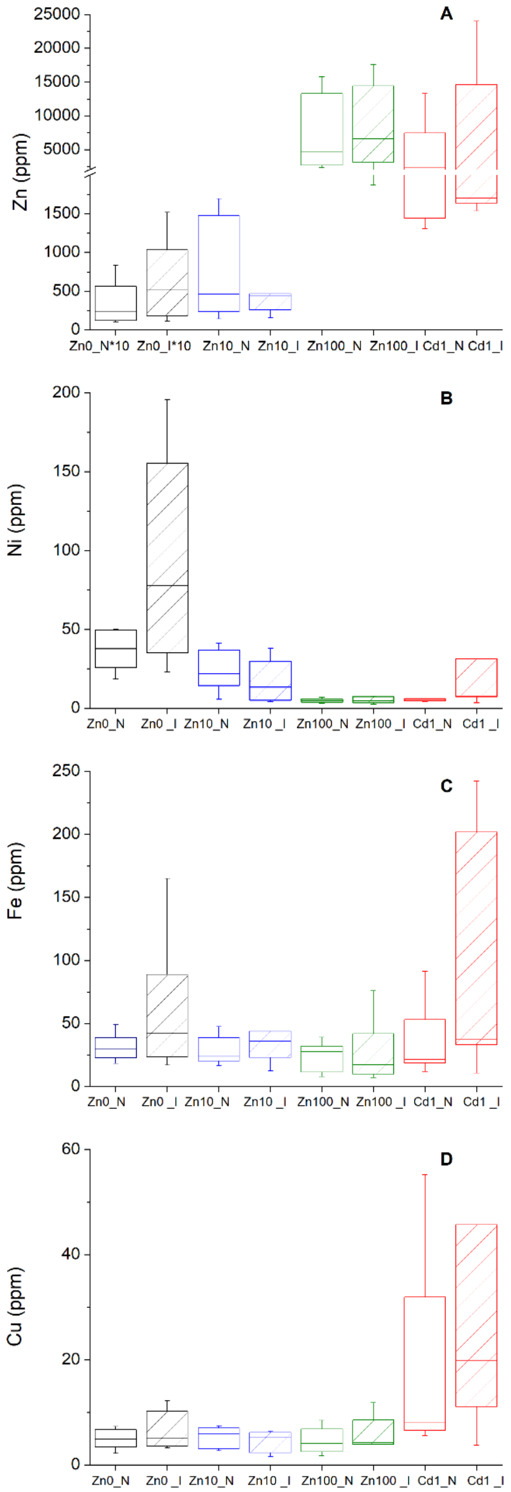

Supplementary Figure S8. Metal content in the leaves of *N. ochroleucum* plants. Zn0, Zn10 and Zn100 refer to Zn concentrations used in the treatments ('0' = 0.01, 10 and 100 µM Zn), Cd1 refers to 1 µM Cd + 10 µM Zn treatment. N-non-infected – empty box plots. I-infected- box plots with stripes. The line presents the median value (n=6-8), box shows the values between 0.25-0.75 percentile, and the bars show whiskers with 1.5 coefficient for outliers. For better presentation, Zn content in Zn0_N and Zn0_I is multiplied by 10.

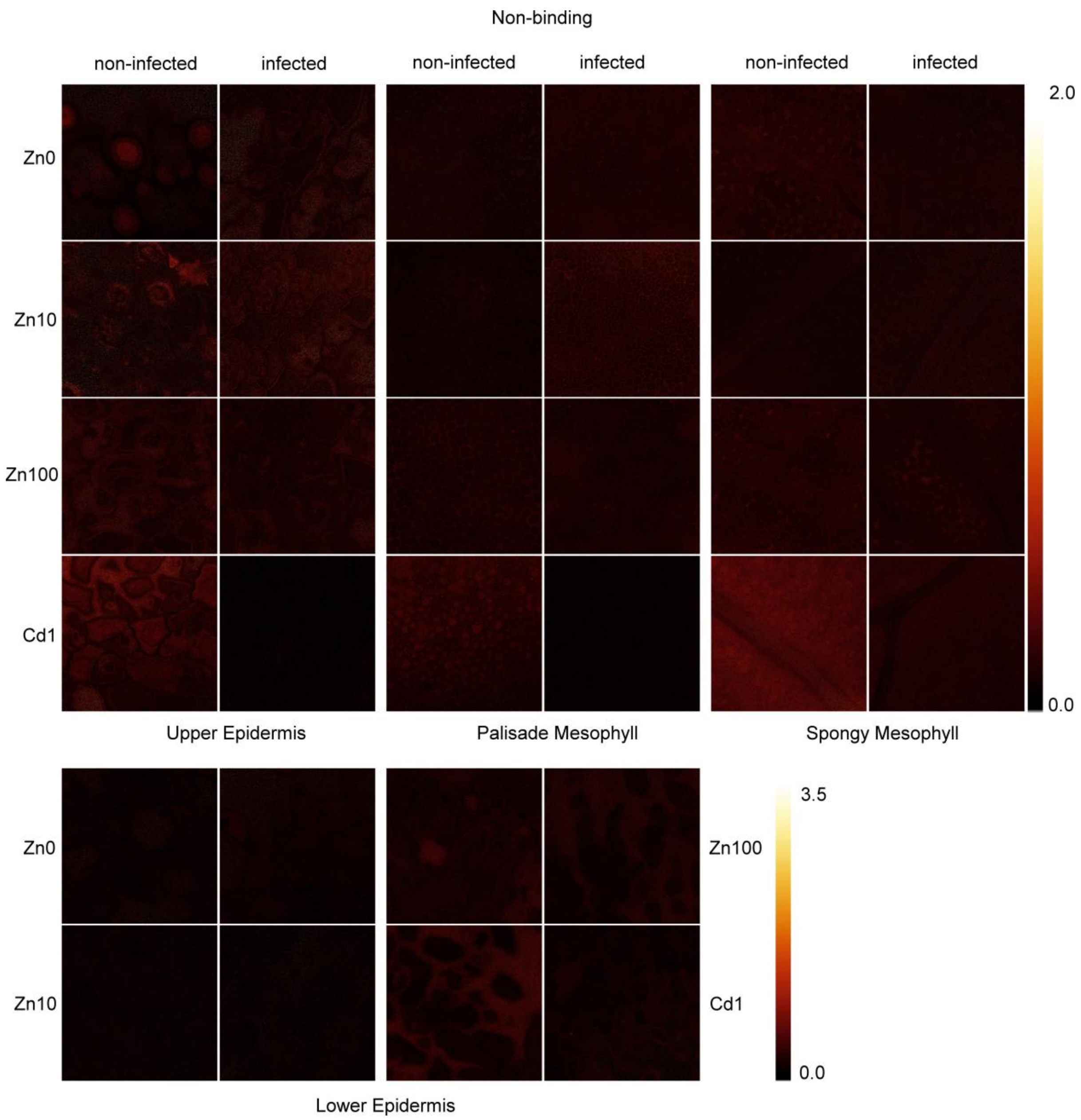

Supplementary Figure S9. Images showing the background signal, measured like cellular expression of the genes of interest, of the non-binding oligo in the leaves of *N. ochroleucum* (non-binding oligo/ GADPH probe fluorescent signal). This was taken as a reference to see the level of non-specific binding of the oligos in the tissue, as described by Küpper et al. (2007b). The scale bars for all genes within the same tissue are shown.
