# Supplementary material for: Interaction Between Zn Deficiency, Toxicity and Turnip Yellow Mosaic Virus Infection in Noccaea ochroleucum
Source: Front Plant Sci. 2020 Jun 5;11:739. doi: 10.3389/fpls.2020.00739 (PMC7290001; doi:10.3389/fpls.2020.00739)
Supplement: Supplementary file 2 [file Table_1.pdf]

Supplementary Table S1. Description of photosynthetic parameters used in the study, according to Stirbet and Govindjee (2011) for OJIP and Genty et al. (1989) for FKM parameters. F_m – maximal chlorophyll fluorescence in dark-adapted state; F_m' – maximal chlorophyll fluorescence in light-adapted and relaxation state; F_o- minimal chlorophyll fluorescence in dark-adapted state. $\Phi_{PSII} = (F_m' - F_t')/F_m')$ and complete NPQ = $(F_m - F_m')/F_m$ were measured from the beginning (i1) to the end (i6) of the 200 s actinic light phase and from the beginning (r1) and the end (r5) of the 200 s dark relaxation phase using 600 ms flash of saturating light as described by Küpper et al. (2007a).

OJIP parameters	
Φ_{Po}	Maximum quantum yield of primary PSII photochemistry
Φ_{ET2o}	Quantum yield of electron transport flux from Q_A to Q_B
Φ_{RE1o}	Quantum yield of electron transport flux until PSI acceptors
J^{abs}/RC	Average absorbed photon flux per PSII reaction center
J_o^{ET2}/RC	Electron transport flux from Q_A to Q_B per PSII
J_o^{Re1o}/RC	Electron transport flux until PSI acceptors
Kautsky parameters	
$F_v/F_m = (F_m - F_o)/F_m$	Photosynthetic efficiency of photosystem II in the dark-adapted state.
Φ_{PSII}_i1	Operating PSII efficiency in irradiation phase 1
Φ_{PSII}_i6	Operating PSII efficiency in irradiation phase 6
Φ_{PSII}_r1	Operating PSII efficiency in relaxation phase 1
Φ_{PSII}_r5	Operating PSII efficiency in relaxation phase 5
NPQ_i1	Nonphotochemical quenching in irradiation phase 1
NPQ_i6	Nonphotochemical quenching in irradiation phase 5
NPQ_r1	Nonphotochemical quenching in relaxation phase 1
NPQ_r5	Nonphotochemical quenching in relaxation phase 5

Supplementary Table S2. Primers used in real time quantitative qPCR and QISH analysis in the present study according to Mishra et al. (2017), Penazova et al. 2016 and Küpper et al. (2007b).

Gene	Description	Primer sequence (5'–3')
GAPDH	*glyceraldehyde 3-phosphate dehydrogenase (cytosolic)*	F- TGCACCACTAACTGCCTTGC<br>R- AAGCACCTTTCCGACAGCCT
18s	18s ribosomal RNA	F- CTGGCGACGCATCATTCAAA<br>R- CTGCCTTCCTTGGATGTGGT
ZNT5	*N. caerulescens (ZIP metal transport family) METAL TRANSPORTER 5*	F-AAGTCCAGACACTGCCAAAG<br>R-TTCGAAGCACTGATGAAACA
MTP1	*N. caerulescens METAL TRANSPORT PROTEIN 1*	F-CACTCTTGCCTTTTCGGTTA<br>R-AAACCCTTTTCGAGCTTTGT
HMA3	*N. caerulescens HEAVY METAL ATPASE 3*	F-ATCAACCTCTGTCGAGCCTAAG<br>R-TCCTTCTCCTGGAAAGTTCTGA
HMA4	*N. caerulescens HEAVY METAL ATPASE 4*	F-CAACTTGTATGGTAGGAGATGGT<br>R-GCTGAGCTCTTCTTGCTAGCTTT
CP	*TYMV Coat protein* (a marker for TYMV infection)	F-CCGGCCCATCACCTCTCACC<br>R-TGGTCGGGAAAGCTGGGGC

QISH		QISH probe sequence, (5'–3')
anti-GAPDH		ACCCTTCAAGTGAGCAGCAGCCTTGTCCTTGTCA
anti-NcZNT5		AATCGACACCTACAATTCCGTGCGCGTGACCATG
anti-NcMTP1		TGGCAAAGGCAGCAACGTCAGAGAGCAAATGTGC
anti-NcHMA4		CACCGCTCTCCACCACCACATTGACCAAACTAGA
anti-NcHMA3		AGGAAGGAGAGAGCAAGCAACACACCAGAAGCCA
NBP	non-binding	GCATTAGCCACGGTAGCGATAGGTAGCATTGGAG

Supplementary table S3. Mann-Whitney U test statistics for significant differences in photosynthetic parameters **for different Zn treatments** in non-infected leaves. Zn0, Zn100 and Zn10+Cd1 were compared to Zn10 as control. Significant differences are emphasized in bold.									
**Photosynthesis parameters**									
	**Zn0_N/Zn10_N**			**Zn10_N/Zn100_N**			**Zn10_N/Cd1_N**		
Kautsky	U	Z	Asymp. Prob>|U|	U	Z	Asymp. Prob>|U|	U	Z	Asymp. Prob>|U|
Fv/Fm	507.5	4.51595	**0.0000**	470	4.94846	**0.0000**	431.5	5.46497	**0.0000**
Φ_{PSII_i1}	480	3.94866	**0.0001**	390	3.12823	**0.0018**	428	5.37557	**0.0000**
Φ_{PSII_i6}	478	3.90742	**0.0001**	376	2.80972	**0.0050**	412	4.96891	**0.0000**
Φ_{PSII_r1}	507	4.50552	**0.0000**	427.5	3.98152	**0.0001**	414	5.01974	**0.0000**
Φ_{PSII_r5}	515	4.67048	**0.0000**	439	4.24302	**0.0000**	422	5.22308	**0.0000**
$NPQ_{\_i1}$	145	-2.94238	**0.0033**	271	0.42175	0.6732	377	4.08796	**0.0000**
$NPQ_{\_i6}$	346.5	1.20371	0.2287	265	0.28523	0.7755	95.5	-3.05692	**0.0022**
$NPQ_{\_r1}$	382.5	1.94907	0.0513	313.5	1.39392	0.1633	150	-1.67348	0.0942
$NPQ_{\_r5}$	410.5	2.52583	**0.0115**	461	4.75577	**0.0000**	297.5	2.06628	**0.0388**

OJIP	**Zn0_N/Zn10_N**			**Zn10_N/Zn100_N**			**Zn10_N/Cd1_N**		
	U	Z	Asymp. Prob>|U|	U	Z	Asymp. Prob>|U|	U	Z	Asymp. Prob>|U|
Φ_{Po}	127	1.3359	0.1816	267.0000	3.5155	**0.0004**	272.0000	4.7842	**0.0000**
Φ_{ET2o}	154	2.1167	**0.0343**	309.0000	4.2517	**0.0000**	294.0000	4.9329	**0.0000**
Φ_{RE1o}	88	-0.7203	0.4713	195.0000	0.8020	0.4226	263.0000	3.8896	**0.0001**
Ψ_{ET2o}	134	1.2440	0.2135	318.0000	4.5241	**0.0000**	294.0000	4.9329	**0.0000**
Ψ_{RE1o}	**78**	**-1.1567**	0.2474	127.5000	-1.2106	0.2261	203.0000	1.8689	0.0616
δ_{Re1o}	85	-0.8511	0.3947	69.5000	-2.9658	**0.0030**	67.0000	-2.6769	**0.0074**
J^{abs}/RC	165.5	-3.3772	**0.0007**	207.0000	-2.6547	**0.0079**	72.0000	-4.6412	**0.0000**
J_o^{ET2}/RC	205	-2.6896	**0.0072**	361.0000	0.0087	0.9931	417.5000	1.9523	0.0509
J_o^{Re1}/RC	176.5	-3.1857	**0.0014**	**290.0000**	-1.2099	0.2263	267.5000	-0.8996	0.3683
J_o^{TR}/RC	164	-3.4034	**0.0007**	319.0000	-0.7050	0.4808	154.5000	-3.0626	**0.0022**
RC/J^{abs}	555	3.3858	**0.0007**	513.0000	2.6547	**0.0079**	558.0000	4.6412	**0.0000**

Supplementary table S4. Mann-Whitney U test statistics for significant differences in photosynthetic parameters **between non-infected and infected leaves within the same Zn or Cd treatment**. Significant differences are emphasized in bold.

**Photosynthesis parameters**												
	**Zn0_N/Zn0_I**			**Zn10_N/Zn10_I**			**Zn100_N/Zn100_I**			**Cd1_N/Cd1_I**		
Kautsky	U	Z	Asymp. Prob>|U|	U	Z	Asymp. Prob>|U|	U	Z	Asymp. Prob>|U|	U	Z	Asymp. Prob>|U|
Fv/Fm	449	4.47053	**0.0000**	318	0.6083	0.54299	250	0.72952	0.46569	225	-0.7769	0.43722
Φ_{PSII_i1}	423	3.87901	**0.0001**	267	-0.4227	0.67251	243	0.54087	0.5886	278	0.36109	0.71803
Φ_{PSII_i6}	426	3.94726	**0.0001**	263.5	-0.49489	0.62068	259	0.95592	0.33911	235	-0.5581	0.57681
Φ_{PSII_r1}	426	3.94765	**0.0001**	299.5	0.22682	0.82056	273	1.29557	0.19512	249	-0.2517	0.8013
Φ_{PSII_r5}	434	4.1294	**0.0000**	325	0.75268	0.45164	277	1.40878	0.1589	233.5	-0.5909	0.55459
$NPQ_{\_i1}$	299	1.05997	0.2892	375	1.79091	0.07331	204	-0.41581	0.67755	425	3.58204	**0.0003**
$NPQ_{\_i6}$	374	2.78531	**0.0054**	436.5	3.06303	**0.00219**	247	0.65639	0.51157	221	-0.865	0.38702
$NPQ_{\_r1}$	340	1.99861	**0.0457**	436.5	3.08864	**0.00201**	253	0.8138	0.41576	257.5	-0.0658	0.94757
$NPQ_{\_r5}$	410	3.59428	**0.0003**	434.5	3.02379	**0.0025**	270	1.24182	0.2143	275	0.29663	0.76675

OJIP	**Zn0_N/Zn0_I**			**Zn10_N/Zn10_I**			**Zn100_N/Zn100_I**			**Cd1_N/Cd1_I**		
	U	Z	Asymp. Prob>|U|	U	Z	Asymp. Prob>|U|	U	Z	Asymp. Prob>|U|	U	Z	Asymp. Prob>|U|
Φ_{Po}	305	3.59401	**0.0003**	244	1.9636	**0.0496**	418	1.76447	0.07765	244	-0.1706	0.8645
Φ_{ET2o}	280	2.87232	**0.0041**	296	2.92812	**0.0034**	407	1.55688	0.1195	291	0.87591	0.3810
Φ_{RE1o}	217	1.05377	0.2920	259	1.91084	0.0560	372	0.89639	0.37005	301	1.10352	0.2698
Ψ_{ET2o}	240	1.7177	**0.0859**	336	4.02771	**0.0001**	391	1.25497	0.20949	377	2.83247	**0.0046**
Ψ_{RE1o}	177	-0.07217	0.9425	231.5	1.15495	0.2481	355	0.57557	0.5649	386	3.03723	**0.0024**
δ_{Re1o}	180	0	1.0000	176	-0.34366	0.7311	348	0.44348	0.65742	273.5	0.47778	0.6328
J^{abs}/RC	118	-3.49503	**0.0005**	310.5	-1.50239	0.1330	210	-2.14194	**0.0322**	281	0.6484	0.5167
J_o^{ET2}/RC	177.5	-2.26828	**0.0233**	378.5	-0.41556	0.6777	304	-0.36801	0.71287	365	2.55955	**0.0105**
J_o^{Re1}/RC	215	-1.49501	0.1350	464	0.93524	0.3497	320	-0.07549	0.93983	361.5	2.47992	**0.0131**
J_o^{TR}/RC	157	-2.69086	**0.0071**	366	-0.61538	0.5383	211	-2.12307	**0.03375**	285	0.7394	0.4597
RC/J^{abs}	458	3.49503	**0.0005**	499.5	1.50239	0.1330	438	2.14189	**0.0322**	223	-0.6484	0.5167

Supplementary table S5. Mann-Whitney U test statistics for significant parameters **for different Zn treatments** in non-infected leaves. Zn0, Zn100 and Zn10+Cd1 were compared to Zn10 as control. Significant differences are emphasized in bold.

**Gene expression**	**Zn0_N/Zn10_N**			**Zn10_N/Zn100_N**			**Zn10_N/Cd1_N**		
	U	Z	Asymp. Prob>|U|	U	Z	Asymp. Prob>|U|	U	Z	Asymp. Prob>|U|
HMA4/GAPDH	20	-1.20774	0.22715	39	1.21514	0.22431	16	-0.51235	0.60841
HMA3/GAPDH	46	1.41778	0.15625	19	-0.98368	0.32527	13	-0.9515	0.34135
MTP1/GAPDH	12	-2.04791	**0.04057**	54	2.95105	**0.00317**	28	1.09789	0.27225
ZNT5/GAPDH	64	3.30816	**9.39E-04**	50	2.48814	**0.01284**	26	0.80512	0.42075
HMA4/18S	35	0.26255	0.7929	36	0.86796	0.38542	13	-0.9515	0.34135
HMA3/18S	54	2.25795	**0.02395**	18	-1.09941	0.27159	14	-0.80512	0.42075
MTP1/18S	34	0.15753	0.87483	49	2.37241	**0.01767**	32	1.68343	0.09229
ZNT5/18S	64	3.30816	**9.39E-04**	52	2.7196	**0.00654**	24	0.51235	0.60841

Supplementary table S6. Mann-Whitney U test statistics for significant differences in gene expression normalized to GAPDH and 18S **between non-infected and infected leaves within the same Zn or Cd treatment**. Significant differences are emphasized in bold.												
**Gene expression**	**Zn0_N/Zn0_I**			**Zn10_N/Zn10_I**			**Zn100_N/Zn100_I**			**Cd1_N/Cd1_I**		
	U	Z	Asymp. Prob>|U|	U	Z	Asymp. Prob>|U|	U	Z	Asymp. Prob>|U|	U	Z	Asymp. Prob>|U|
HMA4/GAPDH	24	-0.78766	0.4309	30	-0.15753	0.87483	42	2.17218	**0.02984**	15	0	1
HMA3/GAPDH	29	-0.26255	0.7929	16	-1.62783	0.10356	23	-0.12778	0.89833	9	-1.0042	0.3153
MTP1/GAPDH	20	-1.20774	0.22715	36	0.36757	0.71319	28	0.38333	0.70148	12	-0.4564	0.64808
ZNT5/GAPDH	21	-1.10272	2.70E-01	21	-1.10272	0.27015	10	-1.78885	0.07364	12	-0.4564	0.64808
HMA4/18S	43	1.10272	0.27015	46	1.41778	0.15625	29	0.5111	0.60928	20	0.82158	0.41131
HMA3/18S	38	0.57762	0.56352	13	-1.94289	**0.05203**	18	-0.76665	0.44329	12	-0.4564	0.64808
MTP1/18S	58	2.67804	**0.00741**	41	0.89268	0.37203	15	-1.14998	0.25015	17	0.27386	0.78419
ZNT5/18S	41	0.89268	0.37203	22	-0.9977	0.31843	2	-2.81106	**0.00494**	11	-0.639	0.52282

Supplementary table S7. Mann-Whitney U test statistics for significant differences in leaf metal content **for different Zn treatments** in non-infected leaves. Zn0, Zn100 and Zn10+Cd1 were compared to Zn10 as control. Significant differences are emphasized in bold.

Metal content	**Zn0_N/Zn10_N**			**Zn10_N/Zn100_N**			**Zn10_N/Cd1_N**		
	U	Z	Asymp. Prob>|U|	U	Z	Asymp. Prob>|U|	U	Z	Asymp. Prob>|U|
Zn	0	-2.92857	0.00341	0	-3.18251	0.00146	7	-2.17218	0.02984
Fe	27	0.78571	0.43203	33	0.52077	0.60252	32	0.89443	0.37109
Cu	20	-0.45185	0.65138	40	0.78766	0.4309	9	-2.14096	0.03228
Ni	33	1.64286	0.10041	54	2.95105	0.00317	38	1.66108	0.0967

Supplementary table S8. Mann-Whitney U test statistics for significant differences in leaf metal content between non-infected and infected leaves within the same Zn or Cd treatment. Significant differences are emphasized in bold.

**Metal content**	**Zn0_N/Zn0_I**			**Zn10_N/Zn10_I**			**Zn100_N/Zn100_I**			**Cd1_N/Cd1_I**		
	U	Z	Asymp. Prob>|U|	U	Z	Asymp. Prob>|U|	U	Z	Asymp. Prob>|U|	U	Z	Asymp. Prob>|U|
Zn	20	-1.20774	0.22715	30	0.63888	0.5229	28	-0.36757	0.71319	13	-0.9515	0.34135
Fe	24	-0.78766	0.4309	18	-0.76665	0.44329	36	0.36757	0.71319	12	-1.0979	0.27225
Cu	25	-0.68264	0.49484	33	0.52077	0.60252	21	-1.10272	0.27015	14	-0.8051	0.42075
Ni	17	-1.5228	0.12781	35	0.75223	0.45191	31	-0.05251	0.95812	10	-1.1368	0.25562
